# HIPK1 Inhibition Protects against Pathological Cardiac Hypertrophy by Inhibiting the CREB‐C/EBP*β* Axis

**DOI:** 10.1002/advs.202300585

**Published:** 2023-04-26

**Authors:** Yihua Bei, Yujiao Zhu, Meng Wei, Mingming Yin, Lin Li, Chen Chen, Zhenzhen Huang, Xuchun Liang, Juan Gao, Jianhua Yao, Petra H. van der Kraak, Aryan Vink, Zhiyong Lei, Yuxiang Dai, Huihua Chen, Yueyang Liang, Joost PG Sluijter, Junjie Xiao

**Affiliations:** ^1^ Institute of Geriatrics (Shanghai University) Affiliated Nantong Hospital of Shanghai University (The Sixth People's Hospital of Nantong) School of Medicine Shanghai University Nantong 226011 China; ^2^ Cardiac Regeneration and Ageing Lab Institute of Cardiovascular Sciences Shanghai Engineering Research Center of Organ Repair School of Life Science Shanghai University Shanghai 200444 China; ^3^ Department of Cardiology Shanghai Tenth People's Hospital Tongji University School of Medicine Shanghai 200072 China; ^4^ Department of Cardiology Shigatse People's Hospital Tibet 857000 China; ^5^ Department of Pathology University Medical Center Utrecht University Utrecht Utrecht 3584 CX The Netherlands; ^6^ Department of Cardiology Laboratory of Experimental Cardiology University Medical Center Utrecht University Utrecht Utrecht 3584 CX The Netherlands; ^7^ Division Lab Central Diagnosis Laboratory Research University Medical Center Utrecht University Utrecht Utrecht 3584 CX The Netherlands; ^8^ Shanghai Institute of Cardiovascular Diseases Zhongshan Hospital Fudan University Shanghai 200032 China; ^9^ School of Basic Medical Science Shanghai University of Traditional Chinese Medicine Shanghai 201203 China; ^10^ UMC Utrecht Regenerative Medicine Center University Medical Center Utrecht Utrecht 3508 GA The Netherlands

**Keywords:** C/EBP*β*, cardiomyocytes, CREB, HIPK1, pathological hypertrophy

## Abstract

Inhibition of pathological cardiac hypertrophy is recognized as an important therapeutic strategy for heart failure, although effective targets are still lacking in clinical practice. Homeodomain interacting protein kinase 1 (HIPK1) is a conserved serine/threonine kinase that can respond to different stress signals, however, whether and how HIPK1 regulates myocardial function is not reported. Here, it is observed that HIPK1 is increased during pathological cardiac hypertrophy. Both genetic ablation and gene therapy targeting HIPK1 are protective against pathological hypertrophy and heart failure in vivo. Hypertrophic stress‐induced HIPK1 is present in the nucleus of cardiomyocytes, while HIPK1 inhibition prevents phenylephrine‐induced cardiomyocyte hypertrophy through inhibiting cAMP‐response element binding protein (CREB) phosphorylation at Ser271 and inactivating CCAAT/enhancer‐binding protein *β* (C/EBP*β*)‐mediated transcription of pathological response genes. Inhibition of HIPK1 and CREB forms a synergistic pathway in preventing pathological cardiac hypertrophy. In conclusion, HIPK1 inhibition may serve as a promising novel therapeutic strategy to attenuate pathological cardiac hypertrophy and heart failure.

## Graphical abstract

Hypertrophic stress‐induced homeodomain interacting protein kinase 1 (HIPK1) is present in the nucleus of cardiomyocytes. HIPK1 inhibition prevents pathological cardiac hypertrophy through inhibiting the cAMP response element binding protein (CREB) phosphorylation at Ser271 and inactivating CCAAT/enhancer‐binding protein β (C/EBPβ). Inhibition of HIPK1 and CREB forms a synergistic pathway in preventing pathological cardiac hypertrophy.

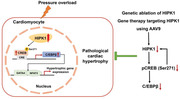

## 1 Introduction

Pathological cardiac hypertrophy is a hallmark of a great variety of cardiovascular diseases (CVDs).^[^ ^1^ ^]^ Along with persisting pathological stresses, the decompensatory hypertrophic growth of myocytes is featured by morphological enlargement and multiple maladaptive responses such as electrical and metabolic dysfunctions, which progressively develops to cardiac remodeling and heart failure.^[^ ^2^, ^3^ ^]^ Unfortunately, effective targeted therapies are still lacking in current daily clinical practice and therefore, new therapies to antagonize pathological cardiac hypertrophy are highly warranted.^[^ ^4^ ^]^

HIPK1 is one of the four evolutionary conserved serine/threonine kinases that belongs to the family of homeodomain interacting protein kinases (HIPKs).^[^ ^5^ ^]^ The HIPK family members are known to respond to various stress signals and play essential roles in a wide range of biological functions such as cell proliferation, differentiation, and apoptosis.^[^ ^6^, ^7^, ^8^ ^]^ Emerging evidence has shown that HIPKs are involved in the developmental processes and cancer development.^[^ ^9^, ^10^, ^11^, ^12^ ^]^ At a molecular level, HIPKs can regulate the activity of many transcription factors as well as cytoplasmic proteins depending on the different cellular context and intracellular locations of HIPKs.^[^ ^13^, ^14^, ^15^, ^16^ ^]^ Nevertheless, current knowledge of the functional roles, as well as, related mechanisms of the HIPK family is still limited for the heart. We previously reported that HIPK1 and HIPK2 are targeted genes of microRNA (miR)‐222 in heart tissues.^[^ ^17^ ^]^ Recently, HIPK2 was found to be essential to maintain basal cardiac function.^[^ ^18^ ^]^ Exercise can downregulate HIPK2 and HIPK2 inhibition was protective against myocardial injury.^[^ ^19^ ^]^ However, the functional role and molecular basis of HIPK1 in the heart have never been reported.

In the current study, we observed that HIPK1 was increased in animal and cellular experimental models of pathological cardiac hypertrophy and also increased in human hypertrophic cardiomyopathy, which prompted us to further examine its functional role and molecular mechanisms. Using mice that were genetically ablated for HIPK1, we demonstrated an obvious antihypertrophic effect with a functional cardiac improvement upon transaortic constriction (TAC)‐induced pathological hypertrophy. Mechanistically, knockdown of HIPK1 in cardiomyocytes attenuated pathological hypertrophy which was associated with inhibition of the cAMP‐response element binding protein (CREB)‐CCAAT/enhancer‐binding protein *β* (C/EBP*β*) axis. We further observed that adeno‐associated virus (AAV)9‐mediated gene therapy that reduced HIPK1 via shRNA approach protected against pathological cardiac hypertrophy and heart failure, and delineated that CREB inhibition was essential to mediate the beneficial effect of reducing HIPK1 in preventing pathological cardiac hypertrophy. These results reveal the functional role and molecular basis of HIPK1 in the heart and indicate HIPK1 inhibition as a promising approach for protecting against pathological cardiac hypertrophy.

## 2 Results

### 2.1 Genetic HIPK1 Ablation Protects against Pathological Cardiac Hypertrophy

**Figure 1:**
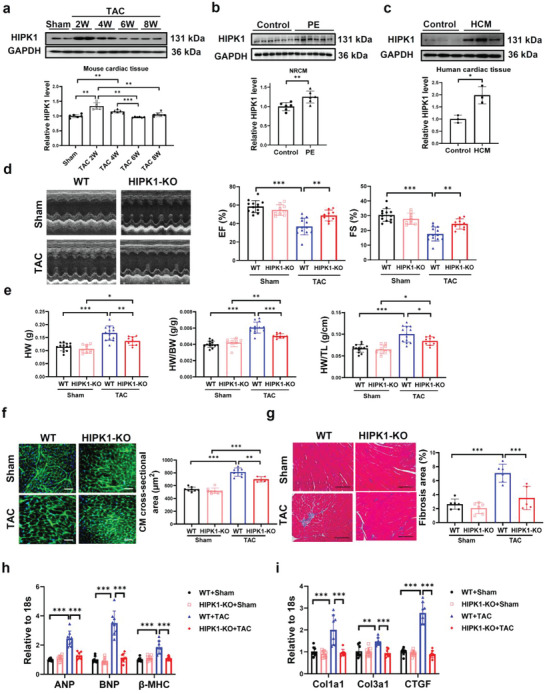
Genetic ablation of HIPK1 prevents TAC‐induced pathological cardiac hypertrophy. a) Western blot for HIPK1 in heart tissues from sham‐operated mice or mice at 2, 4, 6, and 8 weeks post TAC surgery (*n* = 6). b) Western blot for HIPK1 in NRCMs treated with PE or control vehicle (*n* = 6). c) Western blot for HIPK1 in human cardiac tissues from patients with hypertrophic cardiomyopathy and control samples (*n* = 3). d) Echocardiography for LV ejection fraction (EF) and fractional shortening (FS) at 8 weeks post TAC surgery (*n* = 13 for WT mice, *n* = 9 for HIPK1 KO mice). e) Heart weight (HW), heart weight/body weight (HW/BW) ratio, and heart weight/tibia length (HW/TL) ratio of mice (*n* = 9–13). f) Wheat germ agglutinin (WGA) staining for cardiomyocyte (CM) cross‐sectional area (*n* = 8,8,8,5). Scale bar = 50 µm. g) Masson staining for cardiac fibrosis area (*n* = 5–6). Scale bar = 100 µm. h,i) qRT‐PCR for h) ANP, BNP, and *β*‐MHC and i) Col1a1, Col3a1, and CTGF in mice heart tissues after sham or TAC surgery (*n* = 8). Data between two groups were compared by independent‐sample two‐tailed Student's *t*‐test. Data among four groups were compared by one‐way ANOVA test or two‐way ANOVA test followed by Tukey post hoc test. *, *p* < 0.05; **, *p* < 0.01; ***, *p* < 0.001.

To examine whether HIPK1 is regulated in hypertrophic hearts, adult C57BL/6J mice were subjected to TAC surgery to induce pathological cardiac hypertrophy. We observed that HIPK1 was markedly upregulated during myocardial hypertrophic process in mice from 2 to 4 weeks post‐TAC surgery and returned to normal expression level after 6 weeks post‐TAC surgery (**Figure** **1**a). In primary cultured cells, we detected a higher expression of HIPK1 in neonatal rat cardiomyocytes (NRCMs) compared to cardiac fibroblasts (NRCFs; Figure S1a,b, Supporting Information). We further isolated adult mouse cardiomyocytes and noncardiomyocytes (majorly fibroblasts) from sham and TAC mice, and found that HIPK1 was significantly upregulated in cardiomyocytes from the TAC group, indicating a prominent function of HIPK1 in cardiomyocytes (Figure S1c, Supporting Information). Meanwhile, we found that HIPK1 was also upregulated in hypertrophic cardiomyocytes induced by phenylephrine (PE), suggesting a potential role of HIPK1 in cardiomyocyte hypertrophy (Figure 1b). Importantly, we collected human cardiac tissues from patients with hypertrophic cardiomyopathy (HCM) and control samples, and observed a marked upregulation of HIPK1 in HCM group, indicating that HIPK1 was also upregulated in the hypertrophic myocardium in humans (Figure 1c). Collectively, these data consistently demonstrate that HIPK1 is upregulated during pathological myocardial hypertrophy process.

To dissect the functional role of HIPK1 in pathological cardiac hypertrophy in vivo, we subjected HIPK1 KO (Line‐1) mice to TAC model. Analysis of the HIPK1 expression confirmed effective HIPK1 ablation in HIPK1 KO (Line‐1) mice heart tissues (Figure S2a, Supporting Information). Echocardiography and hemodynamic measurement at 4 weeks after TAC showed significantly improved cardiac function, reduced end‐systolic left ventricle (LV) volume, and reduced heart weight in HIPK1 KO mice under TAC stress (Figures S3 and S4, Table S1, Supporting Information). We then measured cardiac function and cardiac remodeling at long term after TAC surgery. Echocardiography demonstrated that HIPK1 KO mice had significantly preserved cardiac function at 8 weeks after TAC‐induced pathological hypertrophy (Figure 1d and Figure S5, Table S2, Supporting Information). Meanwhile, TAC‐induced cardiac remodeling was attenuated in HIPK1 KO mice, as evidenced by significantly reduced heart weight/body weight (HW/BW) ratio and heart weight/tibia length (HW/TL) ratio, myocardial cross‐sectional area, and cardiac fibrosis (Figure 1e–g). These functional and histological improvements in HIPK1 KO animals were accompanied by reduced expression levels of pathological hypertrophy marker genes (ANP, BNP, and *β*‐MHC) and fibrosis‐associated genes (Col1a1, Col3a1, and CTGF) (Figure 1h,i). Using another HIPK1 KO mice line (Line‐2) (Figure S2b, Supporting Information), we confirmed these results having improved cardiac function and reduced myocardial hypertrophy and cardiac fibrosis after TAC surgery in HIPK1 KO mice (Figures S6 and S7, Table S3, Supporting Information). Collectively, genetic HIPK1 removal attenuates TAC‐induced pathological cardiac hypertrophy and cardiac dysfunction.

### 2.2 HIPK1 Knockdown Reduces Pathological Hypertrophy of Neonatal Rat Cardiomyocytes

**Figure 2:**
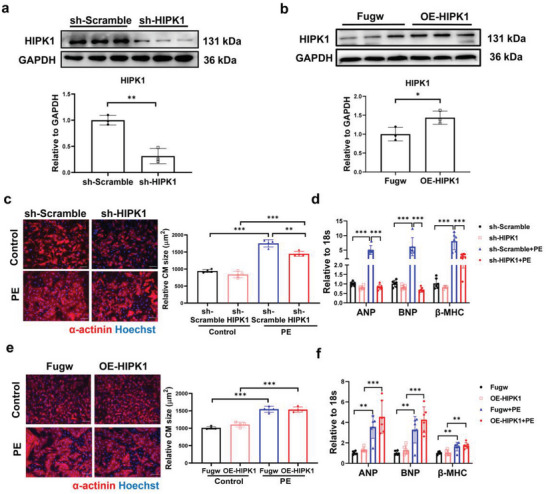
Inhibiting HIPK1 prevents cardiomyocyte hypertrophy. a,b) Western blot for HIPK1 in NRCMs treated with a) sh‐HIPK1 or b) OE‐HIPK1 lentivirus and relative controls (*n* = 3). c,e) Immunofluorescent staining for *α*‐actinin to evaluate cardiomyocyte (CM) size in NRCM treated with c) sh‐HIPK1 or e) OE‐HIPK1 lentivirus upon PE stress (*n* = 4). Scale bar = 50 µm. d,f) qRT‐PCR for ANP, BNP, and *β*‐MHC in NRCM treated with d) sh‐HIPK1 or f) OE‐HIPK1 lentivirus upon PE stress (*n* = 6). Data between two groups were compared by independent‐sample two‐tailed Student's *t*‐test. Data among four groups were compared by two‐way ANOVA test followed by Tukey post hoc test. *, *p* < 0.05; **, *p* < 0.01; ***, *p* < 0.001.

To determine whether HIPK1 could regulate pathological hypertrophy in cardiomyocytes, we first confirmed that lentivirus‐mediated knockdown (sh‐HIPK1) or overexpression (OE‐HIPK1) of HIPK1 could efficiently regulate HIPK1 expression in NRCMs (**Figure** **2**a,b). Immunofluorescent staining for *α*‐actinin demonstrated that HIPK1 knockdown reduced PE‐induced hypertrophy of cardiomyocytes (Figure 2c), accompanied with reduced mRNA expression levels of ANP, BNP, and *β*‐MHC (Figure 2d). HIPK1 overexpression did not further enhance PE‐induced cardiomyocyte hypertrophy (Figure 2e), nor ANP, BNP, or *β*‐MHC levels were regulated by HIPK1 overexpression in PE‐stressed cardiomyocytes (Figure 2f). We suppose that PE‐induced hypertrophic response of cardiomyocytes is too strong to be further enhanced by HIPK1 overexpression, whereas HIPK1 knockdown is sufficient to reduce pathological hypertrophy of cardiomyocytes upon PE stimulation.

### 2.3 HIPK1 Phosphorylates CREB and Activates C/EBP*β* in Cardiomyocytes

**Figure 3:**
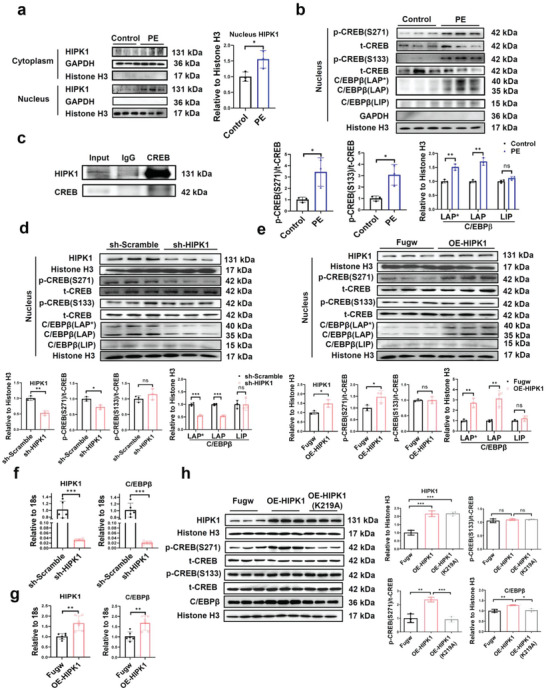
HIPK1 phosphorylates CREB and activates C/EBP*β* in cardiomyocytes. a) NRCMs were treated with PE, and HIPK1 expression was evaluated by Western blot in the cytoplasm and nucleus components, respectively (*n* = 3). b) Western blot for CREB phosphorylation and C/EBP*β* isoform expression in the nucleus component of NRCMs treated with PE (*n* = 3). c) H9C2 cell lysates were incubated with CREB antibody, and co‐immunoprecipitation of CREB and HIPK1 was evaluated by Western blot. d,e) Western blot for HIPK1, CREB phosphorylation, and C/EBP*β* isoform expressions in NRCMs transfected with d) sh‐HIPK1 or e) OE‐HIPK1 lentivirus (*n* = 3). f,g) qRT‐PCR for HIPK1 and C/EBP*β* in NRCMs transfected with f) sh‐HIPK1 (*n* = 5) or g) OE‐HIPK1 (*n* = 6) lentivirus. h) Western blot for HIPK1, CREB phosphorylation, and C/EBP*β* expressions in NRCMs transfected with OE‐HIPK1, OE‐HIPK1 (K219A, kinase‐inactive mutant form of HIPK1 by changing Lysine to Alanine at position 219), and Fugw control plasmids (*n* = 3). Data between two groups were compared by independent‐sample two‐tailed Student's *t*‐test. Data among three groups were compared by one‐way ANOVA test. *, *p* < 0.05; **, *p* < 0.01; ***, *p* < 0.001; ns, not significant.

HIPK1 functions as a serine/threonine‐protein kinase and localizes predominantly in the nucleus.^[^ ^11^ ^]^ HIPK1 was previously reported to induce phosphorylation of the CREB, which is a critical transcription factor that can activate C/EBP*β* transcription.^[^ ^20^, ^21^ ^]^ Moreover, activated C/EBP*β* is involved in pathological cardiac hypertrophy.^[^ ^21^ ^]^ To elucidate the mechanism by which HIPK1 regulates pathological cardiac hypertrophy, we first determined HIPK1 expression in PE‐stressed NRCMs. Our results showed that HIPK1 was significantly upregulated in the nucleus but not in the cytoplasmic components of cardiomyocytes after PE treatment (**Figure** **3**a). We further observed that increased nuclear expression of HIPK1 in PE‐stressed cardiomyocytes was accompanied by increased phosphorylation of CREB at both Ser133 and Ser271 and also by increased protein levels of the liver‐enriched activating protein* (LAP*) and LAP isoforms of C/EBP*β*, while no significant change was found for the liver‐enriched inhibitory protein (LIP) isoform of C/EBP*β* (Figure 3b). These data suggest that activation of CREB may contribute to the functional role of HIPK1 in pathological hypertrophy of cardiomyocytes.

Next, we conducted co‐immunoprecipitation assay in rat H9C2 cardiomyocytes to explore whether HIPK1 could interact with CREB and then induce its phosphorylation. Upon protein precipitation using a CREB antibody, we found a marked presence of HIPK1 compared to normal IgG‐incubated proteins (Figure 3c), indicating an interaction between HIPK1 and CREB. We continued to determine whether and how HIPK1 regulated CREB phosphorylation and C/EBP*β* expression in cardiomyocytes. Using the nuclear components of NRCMs, we detected that knockdown of HIPK1 significantly reduced CREB phosphorylation at Ser271 but not at Ser133; overexpressing HIPK1 had opposite effects (Figure 3d,e). We then analyzed three isoforms of C/EBP*β*,^[^ ^22^, ^23^ ^]^ and showed that knockdown of HIPK1 was able to reduce LAP* and LAP isoforms of C/EBP*β* in the nucleus of cardiomyocytes, while overexpressing HIPK1 increased its LAP* and LAP isoforms; HIPK1 did not alter the LIP isoform of C/EBP*β* (Figure 3d,e). Moreover, HIPK1 positively regulated C/EBP*β* at mRNA level in cardiomyocytes (Figure 3f,g).

To further elucidate whether HIPK1 protein kinase activity is essential for CREB phosphorylation and subsequent C/EBP*β* upregulation, we constructed a kinase‐inactive mutant form of HIPK1 by changing Lysine to Alanine at position 219 (HIPK1‐K219A) and transfected NRCMs with OE‐HIPK1, OE‐HIPK1‐K219A, and Fugw control plasmids, respectively. Our results showed that OE‐HIPK1 plasmids increased CREB phosphorylation at Ser271 and upregulated C/EBP*β* protein level without regulation of CREB phosphorylation at Ser133, while the increased p‐CREB (S271) and C/EBP*β* levels were blunted in NRCMs transfected with OE‐HIPK1‐K219A plasmids (Figure 3h). Collectively, these data demonstrate that pathological stress‐induced HIPK1 can phosphorylate CREB and upregulate C/EBP*β* in cardiomyocytes and that HIPK1 activity is required for CREB phosphorylation at Ser271 and C/EBP*β* upregulation in cardiomyocytes.

### 2.4 HIPK1 Knockdown Reduces Pathological Cardiac Hypertrophy through Inhibiting the CREB‐C/EBP*β* Axis

**Figure 4:**
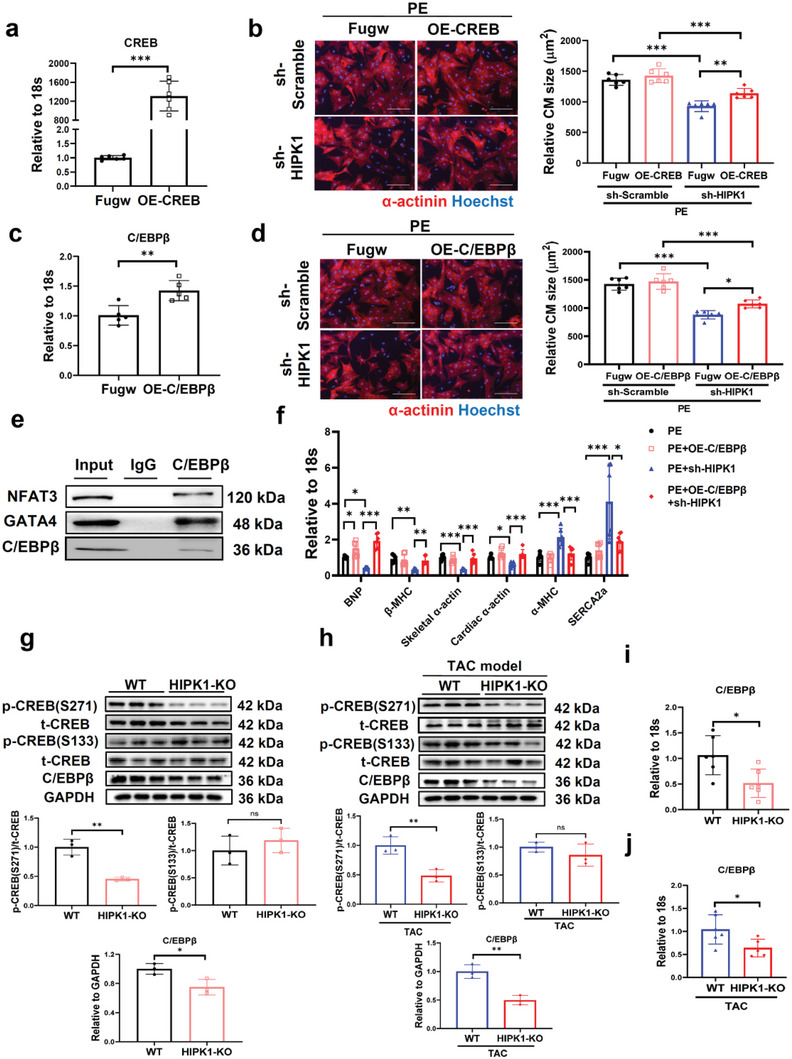
HIPK1 knockdown inhibits pathological hypertrophy of cardiomyocytes through inactivation of the CREB‐C/EBP*β* axis. a) qRT‐PCR for CREB in NRCMs transfected with OE‐CREB plasmid or control vector (*n* = 6). b) Immunofluorescent staining for *α*‐actinin in NRCMs treated with sh‐HIPK1 lentivirus and/or OE‐CREB plasmid (*n* = 6). Scale bar = 100 µm. c) qRT‐PCR for C/EBP*β* in NRCMs transfected with OE‐C/EBP*β* plasmid or control vector (*n* = 5). d) Immunofluorescent staining for *α*‐actinin in NRCMs treated with sh‐HIPK1 lentivirus and/or OE‐C/EBP*β* plasmid (*n* = 6). Scale bar = 100 µm. e) H9C2 cell lysates were incubated with C/EBP*β* antibody, and co‐immunoprecipitation of C/EBP*β* with NFAT3 and GATA4 was evaluated by Western blot. f) qRT‐PCR for genes associated with pathological cardiac hypertrophy in NRCMs (*n* = 6). g,h) Western blot for CREB phosphorylation and C/EBP*β* expressions in heart tissues from WT and HIPK1 KO mice with g) sham or h) TAC operations (*n* = 3). i,j) qRT‐PCR for C/EBP*β* expression in heart tissues from WT and HIPK1 KO mice with i) sham or j) TAC operations (*n* = 5–6). Data between two groups were compared by independent‐sample two‐tailed Student's *t*‐test. Data among four groups were compared by two‐way ANOVA test followed by Tukey post hoc test. *, *p* < 0.05; **, *p* < 0.01; ***, *p* < 0.001.

To assess whether the CREB‐C/EBP*β* axis contributes to the functional role of HIPK1 in pathological cardiac hypertrophy, we co‐treated NRCMs with sh‐HIPK1 and OE‐CREB (or OE‐C/EBP*β*) vectors upon PE stress. Overexpressing CREB or C/EBP*β* was first validated by quantitative reverse transcription polymerase chain reaction (qRT‐PCR, **Figure** **4**a,c). Immunofluorescent staining for *α*‐actinin showed that HIPK1 knockdown significantly attenuated PE‐induced pathological hypertrophy in cardiomyocytes, while this effect was partially reversed by CREB or C/EBP*β* overexpression (Figure 4b,d). However, knockdown of CREB or C/EBP*β* had no additive effect to the antihypertrophic effect of shHIPK1 (Figure S8a–d, Supporting Information). Furthermore, function‐rescue experiment demonstrated that C/EBP*β* downregulation was necessary to mediate the functional role of reducing CREB against pathological hypertrophy (Figure S8e, Supporting Information). Collectively, these data indicate that HIPK1 knockdown prevents pathological hypertrophy of cardiomyocytes at least in part through inhibition of the CREB‐C/EBP*β* axis.

Increased C/EBP*β* was previously reported to interact with transcription factors GATA4 and NFAT;^[^ ^21^, ^24^, ^25^, ^26^ ^]^ the latter ones induce a genetic program associated with pathological cardiac hypertrophy such as upregulated BNP, *β*‐MHC, skeletal *α*‐Actin, and cardiac *α*‐Actin and downregulated *α*‐MHC and SERCA.^[^ ^27^ ^]^ To further elucidate how HIPK1 and C/EBP*β* in cardiomyocytes mediate detrimental effects during pathological hypertrophy, we first demonstrated that C/EBP*β* could interact with GATA4 and NFAT3 in cardiomyocytes using co‐immunoprecipitation assay (Figure 4e). Noteworthy, in PE‐stimulated NRCMs, HIPK1 knockdown led to downregulation of BNP, *β*‐MHC, skeletal *α*‐Actin, and cardiac *α*‐Actin and upregulation of *α*‐MHC and SERCA, however these expression changes were abolished by overexpressing C/EBP*β* (Figure 4f). Consistently, we observed an obvious decrease of CREB phosphorylation at Ser271 but not Ser133 in HIPK1 KO mice in both sham and TAC groups (Figure 4g,h). HIPK1 KO mice also had downregulated C/EBP*β* expression at both protein and mRNA levels in heart tissues (Figure 4g–j). Moreover, we found that TAC‐operated mice had increased skeletal *α*‐Actin and cardiac *α*‐Actin while reduced *α*‐MHC and SERCA expressions in the heart, and that these changes were attenuated in HIPK1 KO animals (Figure S9, Supporting Information). Taken together, these combined data suggest that HIPK1 can regulate the CREB‐C/EBP*β* axis both in vitro and in vivo, and that inhibition of the CREB‐C/EBP*β* axis contributes to the protective effect of suppressing HIPK1 against pathological hypertrophy of cardiomyocytes.

### 2.5 AAV9‐Mediated HIPK1 Knockdown Attenuates Pathological Cardiac Hypertrophy

**Figure 5:**
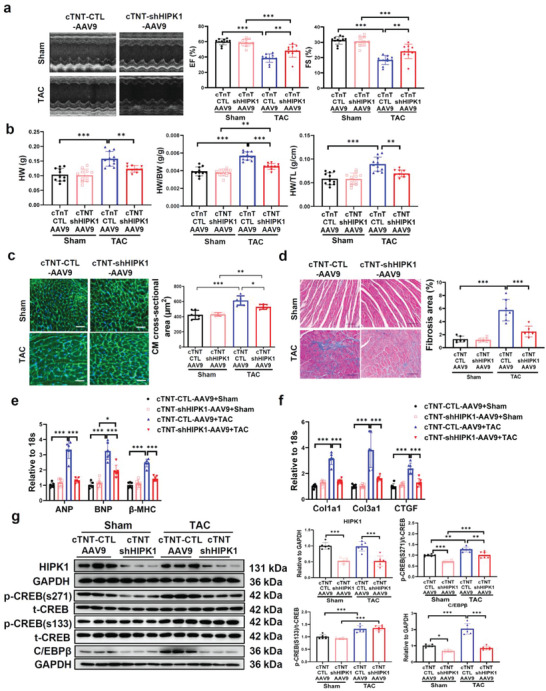
cTnT‐AAV9‐mediated HIPK1 knockdown prevents TAC‐induced pathological cardiac hypertrophy. a) Echocardiography for LV ejection fraction (EF) and fractional shortening (FS) at 6 weeks after TAC surgery (*n* = 10–11). b) Heart weight (HW), heart weight/body weight (HW/BW) ratio, and heart weight/tibia length (HW/TL) ratio of mice (*n* = 10–11). c) Wheat germ agglutinin (WGA) staining for cardiomyocyte (CM) cross‐sectional area (*n* = 6). Scale bar = 50 µm. d) Masson staining for cardiac fibrosis area (*n* = 6–7). Scale bar = 100 µm. e,f) qRT‐PCR for e) ANP, BNP, and *β*‐MHC and f) Col1a1, Col3a1, and CTGF in mice heart tissues after sham or TAC surgery (*n* = 6). g) Western blot for HIPK1, CREB phosphorylation, and C/EBP*β* expressions in mice heart tissues with sham or TAC surgery (*n* = 6). Data among four groups were compared by two‐way ANOVA test followed by Tukey post hoc test. *, *p* < 0.05; **, *p* < 0.01; ***, *p* < 0.001.

After delineating the function and molecular mechanism of HIPK1, we decided to explore the effect of HIPK1 reduction on pathological cardiac hypertrophy via a gene therapy approach. We treated mice via tail vein injections of AAV9 that mediated HIPK1 knockdown driven by a cardiac‐specific troponin promoter (cTnT‐shHIPK1‐AAV9) or control AAV9 vectors (cTnT‐CTL‐AAV9) and subsequently followed 1 week later by TAC surgery to induce pathological cardiac hypertrophy. We first demonstrated that the HIPK1 mRNA level was already significantly reduced at 2 weeks post‐TAC in cTnT‐shHIPK1‐AAV9 injected mice compared to cTnT‐CTL‐AAV9 injected mice (Figure S10a, Supporting Information), and the reduction of HIPK expression was still present in cTnT‐shHIPK1‐AAV9 injected mice at 6 weeks post‐TAC (Figure S10b, Supporting Information). Mice with cTnT‐shHIPK1‐AAV9 gene therapy had a significant improvement of cardiac function (**Figure** **5**a and Figure S11, Table S4, Supporting Information) and a reduction of hypertrophic heart weight after TAC surgery (Figure 5b). TAC‐induced myocardial hypertrophy and cardiac fibrosis were also attenuated upon HIPK1 knockdown (Figure 5c,d), accompanied with reduced expression of pathological hypertrophy marker genes (ANP, BNP, and *β*‐MHC) and fibrosis‐associated genes (Col1a1, Col3a1, and CTGF) (Figure 5e,f). Moreover, treatment with cTnT‐shHIPK1‐AAV9 led to reduced CREB phosphorylation at Ser271 and downregulated C/EBP*β* expression in heart tissues (Figure 5g and Figure S12a, Supporting Information) and also regulated C/EBP*β*’s downstream hypertrophic response genes including downregulating skeletal *α*‐Actin and cardiac *α*‐Actin and upregulating *α*‐MHC and SERCA (Figure S12b, Supporting Information). Interestingly, we found that HIPK1^+/−^ mice were also resistant to pathological cardiac hypertrophy and cardiac dysfunction accompanied with an inhibition of the CREB‐C/EBP*β* axis (Figures S13–S15, Table S5, Supporting Information), supporting that reducing HIPK1, instead of completely suppressing it, was already sufficient to prevent pathological cardiac hypertrophy and cardiac dysfunction. Collectively, these data provide obvious evidence that HIPK1 knockdown exerts a notable beneficial effect to prevent pathological cardiac hypertrophy and suggest that AAV9‐mediated gene therapy is an effective approach to downregulate HIPK1 thus leading to reduced pathological hypertrophy and improved cardiac function in vivo.

### 2.6 Activating CREB Abolishes the Protective Effect of Reducing HIPK1 against Pathological Cardiac Hypertrophy

**Figure 6:**
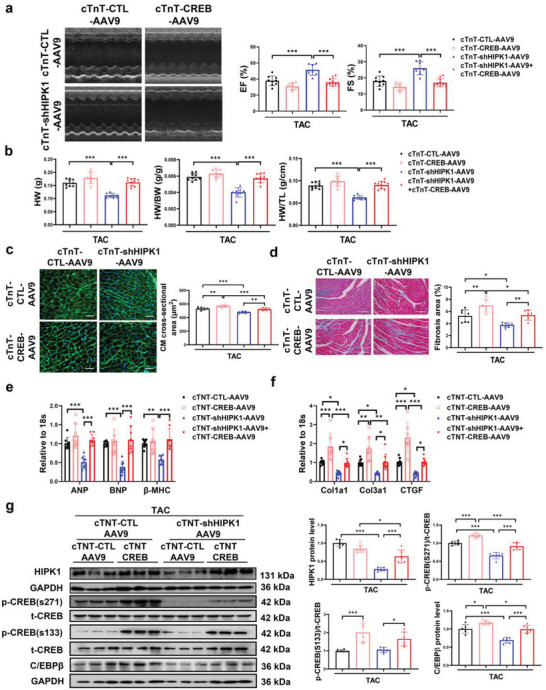
CREB overexpression attenuates the protective effect of HIPK1 knockdown against pathological cardiac hypertrophy. a) Echocardiography for LV ejection fraction (EF) and fractional shortening (FS) at 6 weeks after TAC surgery (*n* = 7–10). b) Heart weight (HW), heart weight/body weight (HW/BW) ratio, and heart weight/tibia length (HW/TL) ratio of mice (*n* = 7–10). c) Wheat germ agglutinin (WGA) staining for cardiomyocyte (CM) cross‐sectional area (*n* = 6). Scale bar = 50 µm. d) Masson staining for cardiac fibrosis area (*n* = 6–8). Scale bar = 100 µm. e,f) qRT‐PCR for e) ANP, BNP, and *β*‐MHC and f) Col1a1, Col3a1, and CTGF in mice heart tissues after TAC surgery (*n* = 7–8). g) Western blot for HIPK1, CREB phosphorylation, and C/EBP*β* expressions in mice heart tissues after TAC surgery (*n* = 6). Data among four groups were compared by two‐way ANOVA test followed by Tukey post hoc test. *, *p* < 0.05; **, *p* < 0.01; ***, *p* < 0.001.

To further elucidate the relationship between HIPK1 and CREB in regulating pathological cardiac hypertrophy, we performed function‐rescue experiment in vivo by treating mice with both cTnT‐shHIPK1‐AAV9 and cTnT‐driven CREB expression AAV9 (cTnT‐CREB‐AAV9) compared to those injected with cTnT‐CTL‐AAV9, cTnT‐CREB‐AAV9, or cTnT‐shHIPK1‐AAV9 alone. Mice were then subjected to TAC surgery at 1 week after AAV9 injections. We first validated that cTnT‐shHIPK1‐AAV9 significantly reduced HIPK1 expression, and that cTnT‐CREB‐AAV9 increased CREB expression at mRNA level in mice heart tissues under TAC stress (Figure S16, Supporting Information). Our results showed that increasing CREB significantly attenuated the protective effect of HIPK1 knockdown in preserving cardiac function and inhibiting myocardial hypertrophy and cardiac fibrosis (**Figure** **6**a–f and Figure S17, Table S6, Supporting Information). Noteworthy, we observed that mice injected with cTnT‐CREB‐AAV9 alone had markedly increased CREB phosphorylation at Ser271 and Ser133 in heart tissues (Figure 6g). Injections of cTnT‐shHIPK1‐AAV9 significantly inhibited CREB phosphorylation at Ser271, while this inhibitory effect was attenuated by co‐injections of cTnT‐shHIPK1‐AAV9 and cTnT‐CREB‐AAV9 (Figure 6g). These results indicate that activating CREB abolishes the protective effect of reducing HIPK1 against pathological cardiac hypertrophy.

Interestingly, we also observed that mice with co‐injections of cTnT‐shHIPK1‐AAV9 and cTnT‐CREB‐AAV9 had upregulated HIPK1 expression compared to those injected with cTnT‐shHIPK1‐AAV9 alone (Figure 6g). We speculated that a synergistic pathway might exist between HIPK1 and CREB. We further demonstrated that increasing CREB upregulated HIPK1, while reducing CREB downregulated HIPK1 at transcriptional level in NRCMs (Figure S18a,b, Supporting Information). Luciferase reporter assay and ChIP assay showed a direct combination and enrichment of CREB to the promoter region of HIPK1 (Figure S18c,d, Supporting Information). Collectively, these data provide in vivo evidence that CREB inhibition is essential to mediate the beneficial effect of targeting HIPK1 in preventing pathological cardiac hypertrophy. In turn, CREB inhibition might also downregulate HIPK1 at transcriptional level, thus forming a beneficial regulation loop in protecting against pathological cardiac hypertrophy.

## 3 Discussion

**Figure 7:**
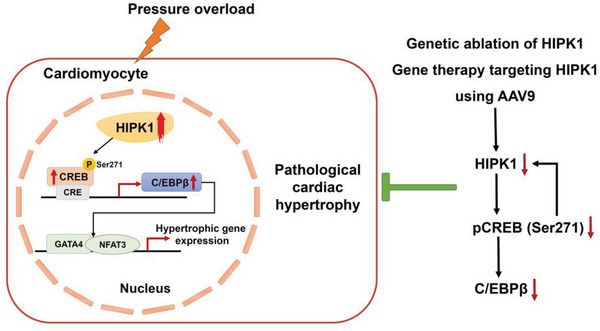
Schematic illustration for the role of HIPK1 in pathological cardiac hypertrophy. Hypertrophic stress‐induced HIPK1 is present in the nucleus of cardiomyocytes, leading to activation of the CREB‐C/EBP*β* axis and subsequent hypertrophic gene expression. HIPK1 inhibition prevents pathological cardiac hypertrophy through inhibiting CREB phosphorylation at Ser271 and inactivating C/EBP*β*. Inhibition of HIPK1 and CREB forms a synergistic pathway in preventing pathological cardiac hypertrophy.

Pathological cardiac hypertrophy is closely related to increased risk of chronic heart failure.^[^ ^28^ ^]^ Although inhibition of pathological cardiac hypertrophy has long been considered as an important therapeutic strategy for heart failure, effective targets that have been applicated to the clinical area are still lacking.^[^ ^4^ ^]^ HIPK1 belongs to the family of homeobox interacting protein kinases, which is known to regulate various central cellular processes.^[^ ^29^, ^30^, ^31^ ^]^ HIPK1 was previously reported as a target gene of miR‐222 in heart tissues, whereas miR‐222 exerted cardioprotective effects.^[^ ^17^ ^]^ However, whether and how HIPK1 could regulate myocardial function directly remains unclear. This is the first study to demonstrate that HIPK1 contributes to pathological cardiac hypertrophy, and that downregulating HIPK1 is beneficial to attenuate pathological hypertrophy, cardiac remodeling, and heart failure. Our study also reveals that reducing HIPK1 can prevent pathological cardiac hypertrophy through inhibition of the CREB‐C/EBP*β* axis. Furthermore, we demonstrate that gene therapy using AAV9‐mediated HIPK1 knockdown is protective against pathological cardiac hypertrophy through inhibiting CREB and that inhibition of HIPK1 and CREB forms a synergistic pathway in preventing pathological cardiac hypertrophy. Collectively, our data identify a novel and important role of HIPK1 in pathological cardiac hypertrophy and suggest that targeting HIPK1 is a promising therapeutic strategy to attenuate pathological cardiac hypertrophy and heart failure (**Figure** **7**).

In the present study, we first observed that HIPK1 was upregulated in cardiomyocytes during the hypertrophic process of TAC‐induced cardiac hypertrophy, and was consistently upregulated in the cellular model of hypertrophic cardiomyocytes upon PE stress. Importantly, HIPK1 was also upregulated in the hypertrophic myocardium in humans. We then examined the development of pathological cardiac hypertrophy using genetic HIPK1 ablation mice. In the experimental TAC model, HIPK1 KO mice manifested markedly reduced pathological myocardial hypertrophy and developed less interstitial fibrotic changes after TAC injury. Meanwhile, HIPK1 ablation significantly preserved cardiac function and reduced ANP, BNP, and *β*‐MHC expressions in hearts after TAC injury. Consistently, HIPK1 knockdown also exerted antihypertrophic effect in PE‐induced cardiomyocyte hypertrophy in vitro model, accompanied with reduced expressions of ANP, BNP, and *β*‐MHC. Collectively, these data for the first time reveal that reducing HIPK1 has an antihypertrophic effect against pathological cardiac hypertrophy.

HIPK1 was originally identified as a nuclear protein kinase but can also be translocated to the cytoplasm upon different stress signals.^[^ ^5^, ^13^, ^31^ ^]^ HIPK1 can function by phosphorylating many different substrates, including transcription factors, enzymatic proteins, and scaffolding proteins.^[^ ^11^, ^32^, ^33^ ^]^ It was previously demonstrated that tumor necrosis factor (TNF‐*α*) can induce deSUMOylation and cytoplasmic translocation of HIPK1, subsequently leading to ASK1‐JNK activation and apoptosis of endothelial cells.^[^ ^13^ ^]^ However, whether and how hypertrophic stress could regulate the cellular location and expression of HIPK1 in cardiomyocytes was unclear. In the present study, we first determined whether the upregulated HIPK1 upon hypertrophic stress was present in the nucleic or cytoplasmic compartment of cardiomyocytes. Our results showed that HIPK1 was markedly upregulated in the nuclear part of hypertrophic NRCMs, while cytoplasmic HIPK1 expression remained at a low level. This indicates that HIPK1 may probably interact with proteins in the nucleus upon stress and thereby modulate subsequent signaling pathways involved in the pathological hypertrophy.

CREB is a nuclear transcription factor whose activation through phosphorylation can lead to transcription of target genes containing the cAMP response element (CRE) sites in their promoters.^[^ ^34^ ^]^ Previous studies reported that HIPK1 can activate CREB through phosphorylation at Ser271 in HEK293 cells, serving as a parallel mechanism with the protein kinase A (PKA)‐induced activation of CREB at Ser133.^[^ ^20^ ^]^ Interestingly, CREB activation has been known to contribute to pathological cardiac hypertrophy.^[^ ^35^ ^]^ Thus, we hypothesized that HIPK1 may activate CREB phosphorylation and contribute to pathological hypertrophy. Our results indicate that CREB phosphorylation levels at both Ser133 and Ser271 were markedly increased in the nuclear fraction of hypertrophic cardiomyocytes, supporting that CREB activation is closely related to hypertrophic stresses.^[^ ^35^, ^36^, ^37^ ^]^ Notably, co‐immunoprecipitation assays provided evidence that HIPK1 could interact with CREB in cardiomyocytes. We further observed that HIPK1 knockdown reduced CREB phosphorylation at Ser271 but not Ser133 in the nucleus component of cardiomyocytes, while HIPK1 overexpression increased CREB phosphorylation at Ser271 without changing the Ser133 phosphorylation site. Our results in cardiomyocytes were consistent with a previous study reporting that HIPK1 can activate CREB through Ser271 phosphorylation in HEK293 cells in vitro,^[^ ^20^ ^]^ and proved that HIPK1‐induced CREB phosphorylation at Ser271 was also present in cardiomyocytes. We further provided evidence that HIPK1 protein kinase activity was required for CREB phosphorylation at Ser271, since the K219A kinase‐dead HIPK1 mutant was no more able to phosphorylate CREB at Ser271 in cardiomyocytes. Compared to other studies reporting CREB phosphorylation at Ser133 during pathological hypertrophy,^[^ ^35^, ^37^ ^]^ our findings of HIPK1‐regulated CREB phosphorylation at Ser271 may provide a novel interventional approach in the development of pathological hypertrophy.

It has been known that CREB activation can promote the transcription of CRE‐regulated C/EBP*β*.^[^ ^38^ ^]^ C/EBP*β* plays important roles in both cardiac physiological hypertrophy and pathological hypertrophy. Reduction of C/EBP*β* has antihypertrophic effect in cellular models of pathological cardiac hypertrophy.^[^ ^21^, ^23^ ^]^ On the other hand, C/EBP*β* was downregulated during exercise‐induced physiological cardiac hypertrophy, and reduction of C/EBP*β* resulted in a cardiac phenocopy of endurance exercise characterized with cardiomyocyte hypertrophy and proliferation.^[^ ^23^ ^]^ Molecules that involved in exercise‐induced physiological cardiac hypertrophy have the potential to brake bad hypertrophy.^[^ ^39^, ^40^, ^41^ ^]^ Reduction of C/EBP*β* has also been shown to protect against pathological hypertrophy and heart failure upon pressure overload in mice.^[^ ^23^ ^]^ Thus, reduced C/EBP*β* at baseline (without pathological hypertrophy stress) can induce a cardiac phenocopy of endurance exercise characterized with cardiomyocyte hypertrophy and proliferation. On the contrary, pathological hypertrophy stress increased C/EBP*β* expression and activity in the heart; in this pathological condition, reduction of C/EBP*β* protects the heart from pathological hypertrophy. Here, we observed for the first time that in HIPK1‐knockdown cardiomyocytes, CREB phosphorylation level at Ser271 was reduced, linked to simultaneous lower C/EBP*β* levels at both mRNA and protein levels. Conversely, overexpression of HIPK1 in cardiomyocytes was able to increase C/EBP*β* levels. Function‐rescue experiments further demonstrated that inhibition of the CREB‐C/EBP*β* pathway was necessary to mediate the antihypertrophic effect of downregulating HIPK1 in PE‐stressed cardiomyocytes. C/EBP*β* can interact with GATA4 and NFAT3 in cardiomyocytes, two critical transcription factors contributing to hypertrophic response gene transcriptions.^[^ ^27^ ^]^ Our results also demonstrated that knockdown of HIPK1 in PE‐stimulated cardiomyocytes leads to reduced C/EBP*β* expression accompanied with a genetic program of gene transcriptions involved in pathological hypertrophy, which was however abolished by overexpressing C/EBP*β*. In animal experimental models, we also found that suppressing HIPK1, either in sham‐ or TAC‐operated mice, could significantly reduce CREB phosphorylation level at Ser271 and downregulate C/EBP*β* expression in the heart. Moreover, TAC‐operated HIPK1 KO animals had reduced BNP, *β*‐MHC, skeletal *α*‐Actin, and cardiac *α*‐Actin expressions and increased *α*‐MHC and SERCA expressions compared to TAC‐operated wild type (WT) animals. Collectively, these findings indicate that targeting HIPK1 can prevent pathological cardiac hypertrophy at least partly through inhibition of the CREB‐C/EBP*β* axis.

In addition to the genetic HIPK1 ablation in mice, we also applied cTnT‐sh‐HIPK1‐AAV9 treatment in TAC animal model, and found that gene therapy that reduced HIPK1 in the heart had similar protective effects against pathological cardiac hypertrophy, as indicated by reduced myocardial hypertrophy, cardiac fibrosis, and cardiac dysfunction. Consistent with the regulation of CREB‐C/EBP*β* axis in HIPK1 KO mice, cTnT‐shHIPK1‐AAV9 treatment also led to reduced CREB phosphorylation at Ser271 but not Ser133, and downregulated C/EBP*β* expression in heart tissues. We also observed that HIPK1 hetero‐knockout mice were resistant to pathological cardiac hypertrophy, further supporting that reducing HIPK1 is sufficient to prevent pathological cardiac hypertrophy. Thus, both genetic ablation and gene therapy targeting HIPK1 exert notable beneficial effects against pathological cardiac hypertrophy via inhibition of the CREB‐C/EBP*β* axis. Last but not at least, CREB was found to positively regulate HIPK1 expression both in heart tissues in vivo and in cardiomyocytes in vitro, suggesting that inhibition of HIPK1 and CREB can form a synergistic pathway in preventing pathological cardiac hypertrophy.

In summary, our data describe the antihypertrophic effect of HIPK1 inhibition in pathological cardiac hypertrophy. Inhibition of the CREB‐C/EBP*β* axis is the molecular basis mediating the myocardial protective effect of reducing HIPK1 against pathological cardiac hypertrophy. Our findings propose that targeting HIPK1 and subsequently inhibiting the CREB‐C/EBP*β* axis may provide a novel therapeutic approach for pathological cardiac hypertrophy and heart failure.

## 4 Experimental Section

### Human Cardiac Tissue

The usage of human cardiac tissue was approved by the institutional committee board of Zhongshan Hospital, Fudan University (no. B2021‐418R) and the study was performed according to the Declaration of Helsinki. Cardiac tissue was obtained from hypertrophic cardiomyopathy (HCM) patients with severe outflow track obstruction undergoing surgical myectomy/septal reduction therapy. Cardiac tissue for normal control was obtained from nonfailing donors with accidental death (traffic accident, trauma, asphyxia, etc.) and with no underlying history of heart disease, which were not suitable for transplant. All participants gave written informed consent in accordance with the Declaration of Helsinki.

### Experimental Animals

8–10 weeks old male C57BL/6J mice were purchased from Beijing Vital River Laboratory Animal Technology Co. Ltd. (Beijing, China) and maintained in specific pathogen‐free (SPF) laboratory animal facility of Shanghai University (Shanghai, China). HIPK1 knockout (KO) mice were generated by using CRISPR/Cas9 technology targeting the 1st exon of HIPK1 genomic DNA (encoding the putative kinase domain).^[^ ^14^ ^]^ Briefly, a frameshift mutation was induced in the 1st exon of HIPK1 genomic DNA, leading to an early termination of translation. Two founders, Founder‐1 (with 13 bp deletion before CTCCTTCTCCCAGCTCC, Figure S19a, Supporting Information) and Founder‐2 (with one G insertion before ACCAGGGCCTCCTTCT, Figure S19b, Supporting Information), were crossed into C57BL/6J mice to generate heterozygous mice, which were then intercrossed to produce HIPK1 KO mice, respectively. Genotypes were confirmed by Southern blotting and DNA sequencing of tail DNA using the following primers: sense 5′‐GCTACACCTGTTCTCACAT‐3′ and antisense 5′‐TCTATGATCTGCACGCTAC‐3′ (Figure S19c–e, Supporting Information). All animal experiments were conducted in accordance with the Guidelines on the use and care of laboratory animals for biomedical research published by the National Institutes of Health (No. 85‐23, revised 1996) and approved by the Committee for the Ethics of Animal Experiments of Shanghai University.

### Mouse Model of TAC‐Induced Pathological Hypertrophy

Pathological cardiac hypertrophy was induced by TAC surgery as previously reported.^[^ ^42^ ^]^ The expression level of HIPK1 was determined in heart tissues and in isolated adult mouse cardiomyocytes and noncardiomyocytes (majorly fibroblasts) from mice heart tissues as well.^[^ ^40^ ^]^ Next, to evaluate the role of genetic ablation of HIPK1 in TAC‐induced pathological cardiac hypertrophy, HIPK1 KO mice and WT littermate controls were subjected to TAC or sham operations. Mice with hetero‐knockout of HIPK1 (HIPK1^+/−^) were also evaluated for functional and structural assessments after TAC surgery. To investigate the biological interventions targeting HIPK1 in pathological cardiac hypertrophy, mice were treated with tail vein injections of AAV9 (HANBIO, Shanghai, China) that mediated HIPK1 knockdown driven by a cardiac‐specific troponin promoter (cTnT‐shHIPK1‐AAV9) or control AAV9 vectors (cTnT‐CTL‐AAV9) at a dose of 10^12^ viral genome particles per mouse. To further investigate whether CREB overexpression influenced the function of HIPK1 knockdown in pathological cardiac hypertrophy, mice were injected via tail vein with both cTnT‐shHIPK1‐AAV9 and cTnT‐driven CREB expression AAV9 (cTnT‐CREB‐AAV9) compared to those injected with cTnT‐CTL‐AAV9, cTnT‐CREB‐AAV9, or cTnT‐shHIPK1‐AAV9 alone (HANBIO, Shanghai, China). 1 week after AAV9 injections, mice were subjected to sham operation or TAC surgery to induce pathological cardiac hypertrophy. From 6 weeks after TAC surgery, echocardiography was performed to evaluate cardiac function, and mice were sacrificed at 6 or 8 weeks after TAC surgery. The heart weight, heart weight/body weight (HW/BW) ratio, and heart weight/tibia length (HW/TL) ratio were determined for each mouse. Heart tissues were embedded into paraffin or OCT or snap frozen and conserved at −80 °C for further examinations.

### Primary Cell Isolation, Culture, and Treatment

Primary NRCMs and NRCFs were isolated from Sprague–Dawley (SD) rats at the age of 1–3 days using Collagenase II (Gibco 17101015)/Pancreatin from porcine pancreas (Sigma P3292) digestion and Percoll (GE healthcare 17‐0891‐01) gradient centrifugation method as previously reported.^[^ ^43^ ^]^ HIPK1 expression abundance in NRCMs and NRCFs were determined using qRT‐PCR as described later. Isolated NRCMs were maintained in 4.5 g L^−1^ glucose‐containing Dulbecco's modified Eagle's medium (DMEM, Corning 10‐013‐CVR) supplemented with 10% horse serum and 5% fetal bovine serum for functional experiments. To study the downstream molecular regulations by HIPK1 in cardiomyocytes, NRCMs were infected with HIPK1 overexpression (OE‐HIPK1) or HIPK1 knockdown (sh‐HIPK1) lentivirus for 72 h followed by assessment of CREB phosphorylation and C/EBP*β* expression as described later. To determine whether HIPK1 protein kinase activity is essential for CREB phosphorylation in cardiomyocytes, a kinase‐inactive mutant form of HIPK1 was constructed by changing Lysine to Alanine at position 219 (HIPK1‐K219A) using the TaKaRa MutanBEST Kit,^[^ ^44^ ^]^ and then transfected NRCMs with OE‐HIPK1, OE‐HIPK1‐K219A, and Fugw control plasmids using Lipofectamine 2000 (Invitrogen), respectively.

Pathological hypertrophy of cardiomyocytes was induced by incubating NRCMs with PE (100 × 10^−6^ m, Tocris 2838) for 48 h. For the functional role of HIPK1 in cardiomyocyte hypertrophy, NRCMs were infected with OE‐HIPK1 or sh‐HIPK1 lentivirus for 72 h and stressed with PE treatment for 48 h. To determine whether the CREB‐C/EBP*β* axis was involved in the functional role of HIPK1 in pathological hypertrophy, NRCMs were co‐transfected with CREB or C/EBP*β* overexpression plasmid, simultaneously treated with sh‐HIPK1 lentivirus under PE stress. Moreover, NRCMs were co‐transfected with CREB or C/EBP*β* siRNA, simultaneously treated with sh‐HIPK1 lentivirus under PE stress. Function‐rescue experiment of CREB and C/EBP*β* was performed by co‐transfection of CREB siRNA and C/EBP*β* overexpression plasmid in NRCMs under PE stress. 48 h after treatment, the cells were collected for further experiments.

### Echocardiography

Mice cardiac function was measured from 6 weeks after TAC or sham operation using Vevo 2100 (FUJIFILM Visual Sonics Inc.). Mice were anesthetized with 1.5% isoflurane with spontaneous ventilation. Left ventricular systolic function was measured by 2D M‐mode imaging from the long‐axis view of LV at the papillary muscle level. LV ejection fraction (EF, %) and fractional shortening (FS, %) were calculated and presented for cardiac function.

### Hemodynamic Measurement

Cardiac hemodynamics was measured at 4 weeks after TAC by pressure‐volume loop as previously reported.^[^ ^45^ ^]^ Briefly, mice were anesthetized with isoflurane and mechanically ventilated followed by chest opening. The LV was inserted by a 1.2 F pressure–volume conductance catheter (Scisence, Ontario, Canada) through apex approach. A total of 10–20 steady pressure–volume loops were collected and then analyzed by LabScribe2 software (iWorx, Dover, NH, USA). The maximum LV volume (*V*_max_), minimum LV volume (*V*_min_), end‐systolic volume (ESV) and end‐diastolic volume (EDV) were quantified to evaluate LV volume during pathological cardiac hypertrophy.

### Histology for Myocardial Hypertrophy and Cardiac Fibrosis

For myocardial hypertrophy analysis, 5 µm thick cardiac cryosections were stained with wheat germ agglutinin (WGA, Sigma L4895a) to determine the myocardial cross‐sectional area. For cardiac fibrosis analysis, 5 µm thick cardiac paraffin sections were stained with Masson's Trichrome (Solarbio, G1340) to determine the percentage of fibrotic area to total area. Images were analyzed using Image J software 1.50i (NIH, USA).

### Immunofluorescent Staining for *α*‐Actinin

48 h after treatment, NRCMs were fixed with 4% paraformaldehyde (PFA), and then permeabilized with 0.5% Triton X‐100 in phosphate‐buffered saline at room temperature. Cells were blocked with 5% bovine serum albumin, and then incubated with primary antibody mouse anti‐*α*‐actinin (Sigma A7811) at 4 °C overnight. Next, cells were incubated with Cy3‐labeled secondary antibody for 2 h at room temperature, and then counterstained with Hoechst. Images were taken using fluorescence microscope (Leica DMi8) and analyzed with Image J software 1.50i (NIH, USA) for determination of the cell size of *α*‐actinin‐positive cardiomyocytes.

### Co‐Immunoprecipitation

Rat H9C2 cells were lysed with cell lysis buffer (Merck C2978) supplemented with protease inhibitor cocktail (Roche RD0469315) for 30 min on ice. The cell lysates were centrifuged at 14 000 rpm for 20 min at 4 °C and diluted to 2 mg mL^−1^. The protein A+G agarose beads (Roche 11719416001, 20 µL per sample) were incubated with CREB antibody (Abclonal A11989) or C/EBP*β* antibody (Abcam ab220813) and Normal Rabbit IgG antibody (Millipore, 4 µg per sample) at 4 °C for 2 h with rotation, respectively. After washed three times with cell lysis buffer, the protein A+G agarose beads coupled with CREB (or C/EBP*β*) antibody or normal IgG were further incubated with the cell lysates (300 µL, 2 mg mL^−1^) at 4 °C overnight with rotation. After washed five times with cell lysis buffer, the beads‐bounded proteins were dissociated in 2x loading buffer and heated at 95 °C for 10 min with vortex every 5 min. The identification of CREB protein with associated HIPK1 protein and the identification of C/EBP*β* protein with associated NFAT3 or GATA4 protein were detected by Western blot, respectively.

### Luciferase Reporter Assay

The regulation between CREB and HIPK1 was analyzed by luciferase reporter assay. Briefly, the promoter sequence of HIPK1 was cloned into PGL3‐basic vector. After transfection of OE‐CREB plasmid together with PGL3‐basic vector or HIPK1 promoter containing PGL3 vector in HEK293 cells, luciferase reporter assay was performed using Beyotime Dual‐Lumi Luciferase Reporter Gene Assay Kit (Beyotime RG088). The sequences used for HIPK1 promoter region were as follows: HIPK1‐promoter (175‐580)‐F: GACGCGT ATCACTCGCTGGATGACATTCC, HIPK1‐promoter (175‐580)‐R: CAAGCTT ATCCGGCGGAGGACCTAGAA; HIPK1‐promoter (580‐1000)‐F: GACGCGTTCCGACTGACCGCAGACC, HIPK1‐promoter (580‐1000)‐R: CAAGCTTAAAGGAAGTCACCCTGCCCG; HIPK1‐promoter (175‐1000)‐F: GACGCGTATCACTCGCTGGATGACATTCC, HIPK1‐promoter (175‐1000)‐R: CAAGCTTAAAGGAAGTCACCCTGCCCG.

### ChIP‐PCR Assay

ChIP‐PCR assay was performed to determine the enrichment of CREB to the promoter region of HIPK1 in AC16 cells using SimpleChIP Enzymatic Chromatin IP Kit (Cell Signaling Technology, #9003). Briefly, 4 × 10^6^ AC16 cells were cross‐linked with 37% formaldehyde and 1.5 m glycine. The DNA fragments were extracted using nuclei lysis buffer and treated by ultrasonic fragmentation. The supernatant was incubated with IgG antibody (Cell Signaling Technology, #2729) or CREB antibody (Abclonal, A11989) at 4 °C overnight. The 2% supernatant was taken as input and conserved at 4 °C. Each immunoprecipitation (IP) sample was added with Protein G Magnetic Beads and incubated at 4 °C for 2 h. After centrifugation, the precipitant was repeatedly washed and then added with ChIP elution buffer followed by heating at 65 °C overnight. Finally, DNA was extracted using Spin Columns and then examined by qRT‐PCR. The primer sequences used were as follows: ChIP‐HIPK1‐F1: 5’‐GCTCCCGCTACTTCTAGGTC‐3’, ChIP‐HIPK1‐R1: 5’‐AGCAAGATTGGGCCTATCCG‐3’; ChIP‐HIPK1‐F2: 5’‐TAGGTCCTCCGCCGGATTT‐3’, ChIP‐HIPK1‐R2: 5’‐GGAGACAGGCCAAAGTAGCC‐3’; ChIP‐HIPK1‐F3: 5’‐TACAGCCCACCTCTTGCATC‐3’, ChIP‐HIPK1‐R3: 5’‐CAGTTTTCGTGGCCCCCATT‐3’.

### Western Blot

Cardiac tissues or NRCMs were homogenized and lysed with radioimmunoprecipitation assay lysis buffer (KeyGEN BioTECH) supplemented with protease and phosphatase inhibitor tablet (Thermo Scientific) and 1% phenylmethylsulfonyl fluoride (KeyGEN BioTECH) for protein extraction. For analysis of HIPK1, CREB phosphorylation, and C/EBP*β* expressions in the different components of NRCMs, nuclear and cytoplasmic proteins were extracted from NRCMs in the presence or absence of PE stress using Nuclear and Cytoplasmic Protein Extraction Kit (KeyGEN BioTECH). Equal quantities of extracted proteins were separated by sodium dodecyl sulfate‐polyacrylamide gel electrophoresis gels and transferred to polyvinylidene difluoride membranes. After blocked with 5% nonfat milk, membranes were incubated with primary antibodies at 4 °C overnight, including HIPK1 (Abcam, ab152109), phospho‐CREB (S271) (Abcam, ab119711), phospho‐CREB (S133) (Abcam, ab32096), total‐CREB (Abcam, ab32515), C/EBP*β* (Proteintech, 23431‐1‐AP), C/EBP*β* isoforms (LAP^*^, LAP, LIP, BioLegend, 606202), GATA4 (Affinity, AF5245), and NFAT3 (Affinity, DF8682). The immunoblots were then incubated with appropriate horseradish peroxidase‐conjugated secondary antibodies and visualized using ECL Kit (Thermo Pierce). GAPDH (Bioworld, AP0063) was used as loading control. Specifically, Histone H3 (Abclonal, A2348) was used as loading control for the nuclear proteins. For determination of the CREB phosphorylation levels, membranes were first incubated with phospho‐CREB antibody, and then stripped and incubated with total‐CREB antibody. The protein band density was determined using Image J software 1.50i (NIH, USA). All the protein bands for one target protein were separately framed with rectangles of the same size on a membrane for analysis of band intensity.

### qRT‐PCR

Total RNA was extracted from heart tissues, NRCMs, or NRCFs using RNAiso reagent (TaKaRa). Reverse transcription was performed using RevertAid First Strand cDNA Synthesis Kit (Thermo Scientific). The cDNA was then subjected to qRT‐PCR using appropriate primers and Takara SYBR green in Roche LightCycler 480 PCR System. The primer sequences used are listed in Table S7 in the Supporting Information.

### Statistical Analysis

All statistical data were analyzed with SPSS software Version 20.0 and shown as mean ± standard deviation (SD) using GraphPad Software Version 8.3.0. An independent‐sample *t*‐test (two‐tailed) was used for statistical comparisons between two groups. Two‐way analysis of variance (ANOVA) test followed by Tukey's post hoc test or one‐way ANOVA test was used for statistical comparisons among three or more than three groups as appropriate. A *p*‐value <0.05 was considered as significant.

## Conflict of Interest

The authors declare no conflict of interest.

## Author Contributions

Y.B., Y.Z., M.W., M.Y., L.L., C.C., Z.H., X.L., J.G., and J.Y. performed the experiments and analyzed the data. P.H.v.d.K., A.V., and Z.L. revised the manuscript and polished the language. Y.D. collected human cardiac tissues from patients. H.C. and Y.L. performed hemodynamic measurements. J.P.G.S. helped perform the experiments, provided technical assistance, and revised the manuscript. J.X. designed the experiments and wrote the manuscript.

## Supporting information

Supporting Information

## Data Availability

The data that support the findings of this study are available from the corresponding author upon reasonable request.
