# Supplementary material for: HIPK1 Inhibition Protects against Pathological Cardiac Hypertrophy by Inhibiting the CREB‐C/EBPβ Axis
Source: Adv Sci (Weinh). 2023 Apr 26;10(18):2300585. doi: 10.1002/advs.202300585 (PMC10288234; doi:10.1002/advs.202300585)
Supplement: Supplementary file 1 — Supporting Information [file ADVS-10-2300585-s001.pdf]

ADVANCED SCIENCE
Open Access

Supporting Information

for *Adv. Sci.*, DOI 10.1002/advs.202300585

HIPK1 Inhibition Protects against Pathological Cardiac Hypertrophy by Inhibiting the CREB-C/EBPβ Axis

Yihua Bei, Yujiao Zhu, Meng Wei, Mingming Yin, Lin Li, Chen Chen, Zhenzhen Huang, Xuchun Liang, Juan Gao, Jianhua Yao, Petra H. van der Kraak, Aryan Vink, Zhiyong Lei, Yuxiang Dai, Huihua Chen, Yueyang Liang, Joost PG Sluijter and Junjie Xiao**

Supplementary Materials for

HIPK1 inhibition protects against pathological cardiac hypertrophy by inhibiting the CREB-C/EBPβ axis

Yihua Bei*, Yujiao Zhu, Meng Wei, Mingming Yin, Lin Li, Chen Chen, Zhenzhen Huang, Xuchun Liang, Juan Gao, Jianhua Yao, Petra H van der Kraak, Aryan Vink, Zhiyong Lei, Yuxiang Dai, Huihua Chen, Yueyang Liang, Joost PG Sluijter, Junjie Xiao*

Correspondence to: Junjie Xiao (E-mail: junjiexiao@shu.edu.cn) and Yihua Bei (E-mail: beiyh36@shu.edu.cn).

Supplementary Figures

Figure S1

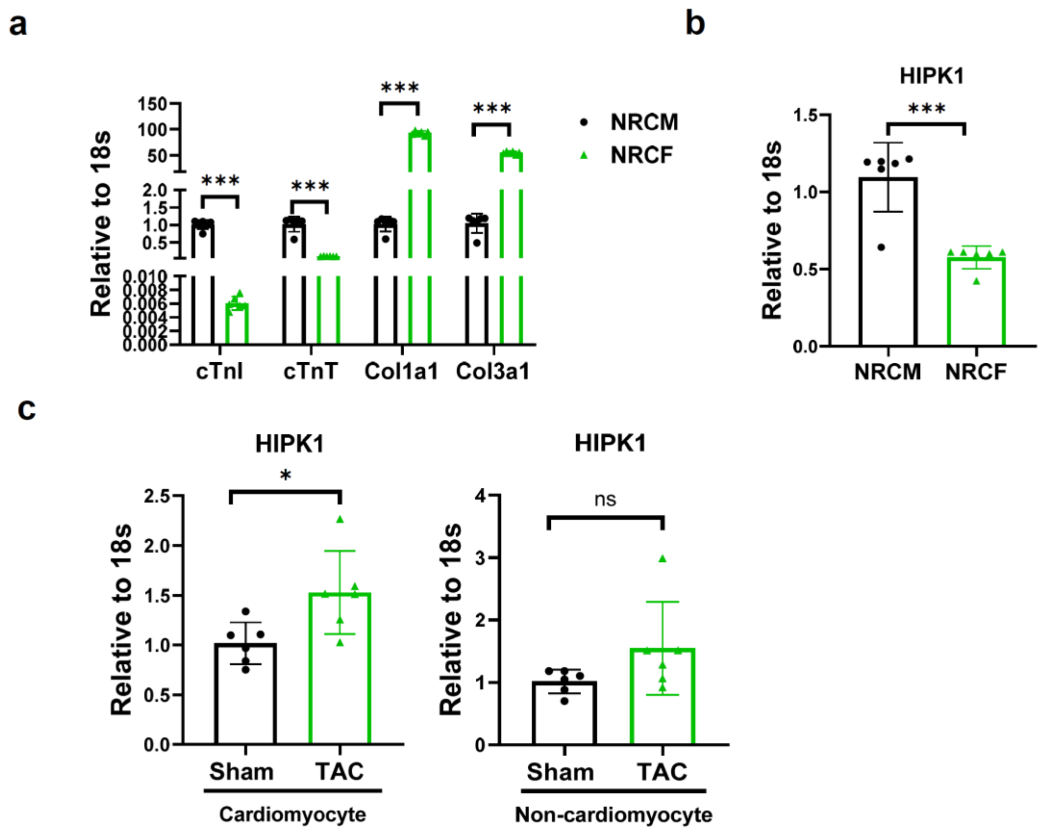

Figure S1. HIPK1 expression in primary cardiomyocytes and non-cardiomyocytes isolated from neonatal rat or adult mice hearts. a and b, qRT-PCR for cTnI, cTnT, Col1a1, Col3a1 **(a)** and HIPK1 **(b)** in isolated neonatal rat cardiomyocytes (NRCM) or fibroblasts (NRCF) (n=6). **c,** qRT-PCR for HIPK1 in isolated adult mouse cardiomyocytes *versus* non-cardiomyocytes (majorly fibroblasts) from hearts post sham operation or transaortic constriction (TAC)-induced pathological cardiac hypertrophy (n=6). Data between 2 groups were compared by independent-sample two-tailed Student's t-test. *, P<0.05; ***, P<0.001; ns, not significant.

Figure S2

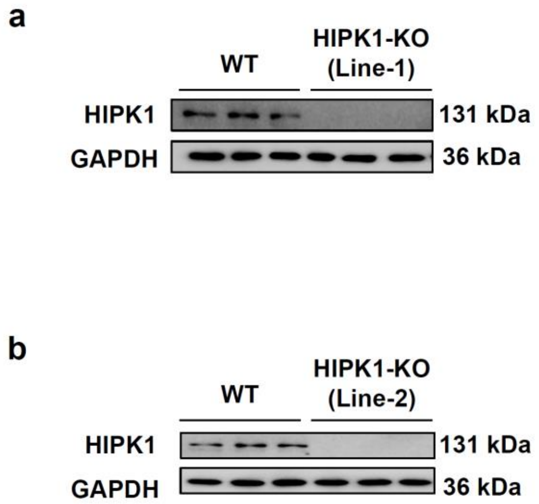

Figure S2. HIPK1 knockout (KO) mice have ablated HIPK1 expression in heart tissues. a and b, Western blot for HIPK1 in heart tissues to confirm HIPK1 ablation in HIPK1 KO mice (Line-1) **(a)** and HIPK1 KO mice (Line-2) **(b)** (n=3).

Figure S3

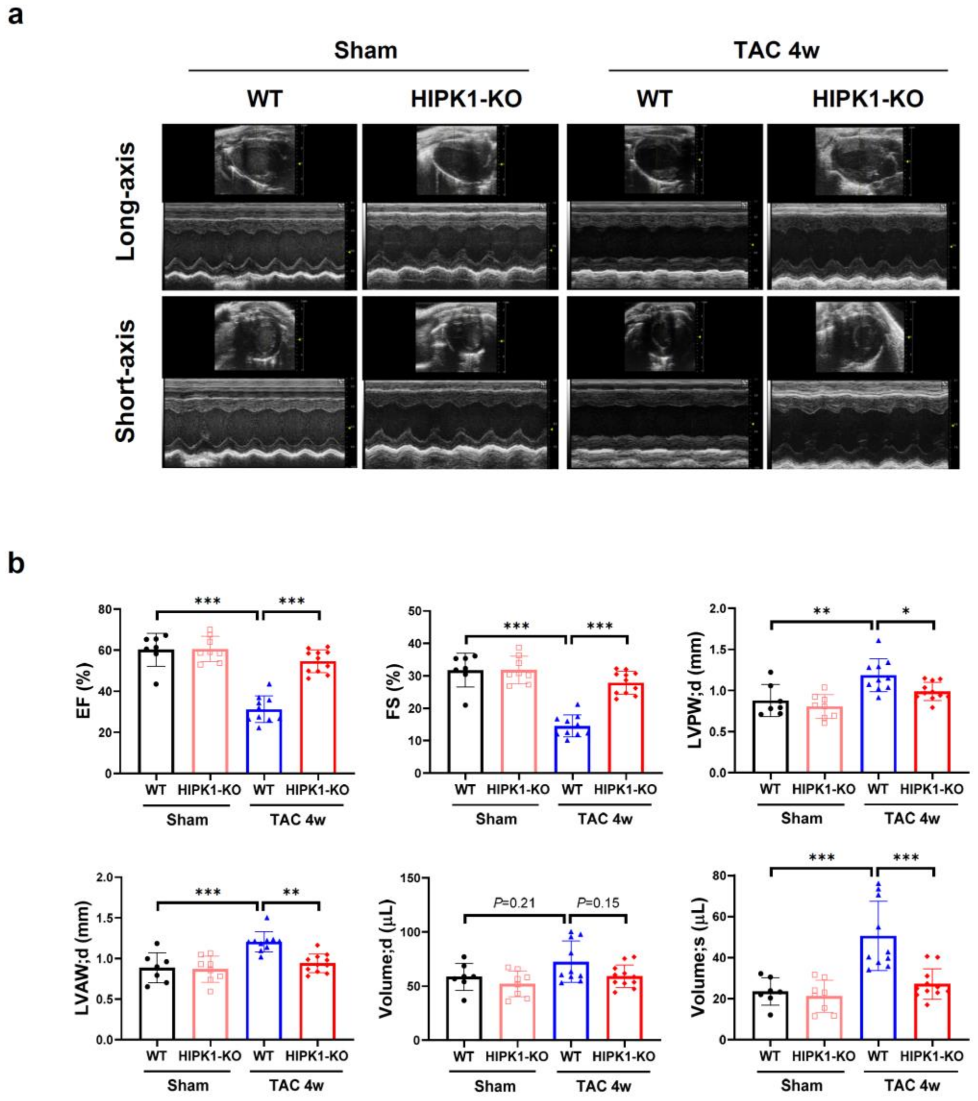

Figure S3. HIPK1 knockout (Line-1) mice have improved cardiac function at 4 weeks after transaortic constriction (TAC) surgery. a, Echocardiography for left ventricle (LV) systolic function of mice with representative images showing the long-axis view and the corresponding short-axis view of LV at the papillary muscle level. **b,** Statistical analysis of LV ejection fraction (EF) and fractional shortening (FS), end-diastolic LV posterior wall thickness (LVPW;d) and anterior wall thickness (LVAW;d), and end-diastolic LV volume (Volume;d) and end-systolic LV volume (Volume;s) according to measurements of three consecutive cardiac cycles from the long-axis view of LV at the papillary muscle level (n=7-11). Data among 4 groups were compared by two-way ANOVA test followed by Tukey post hoc test. *, P<0.05; **, P<0.01; ***, P<0.001.

Figure S4

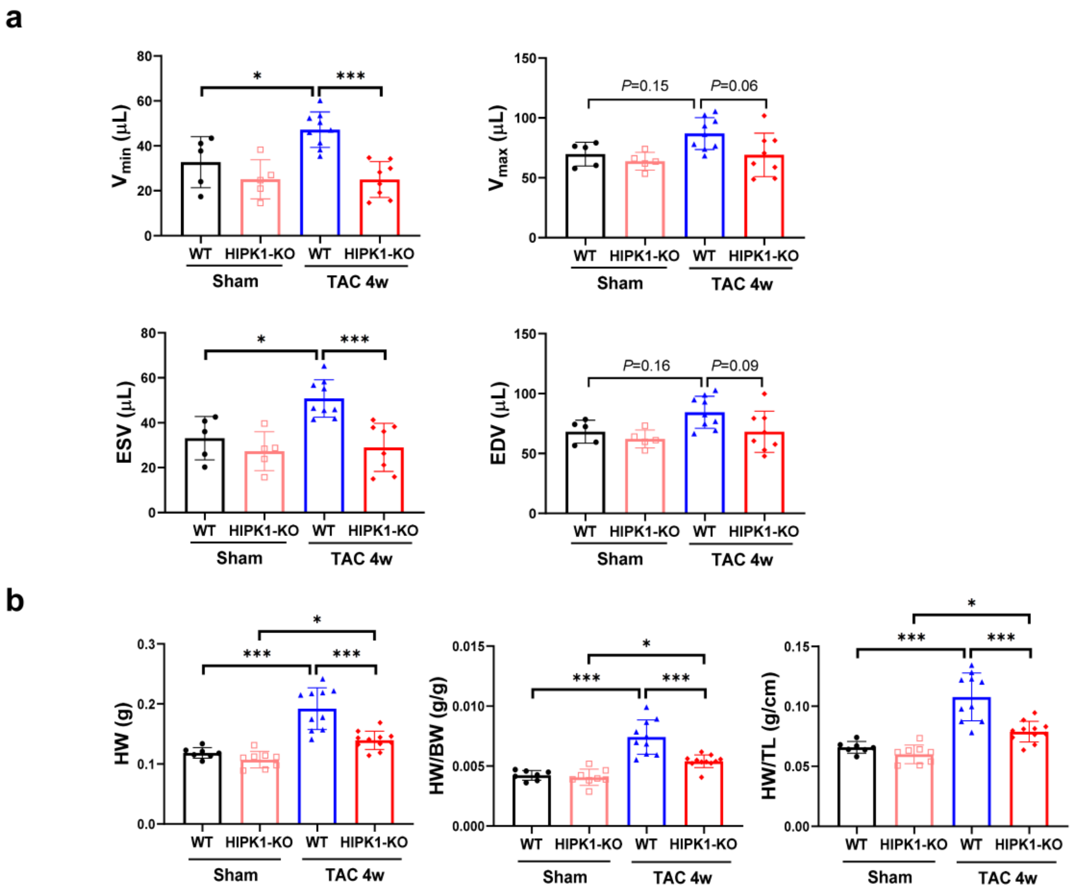

Figure S4. HIPK1 knockout (Line-1) mice have reduced left ventricle volume and heart weight at 4 weeks after transaortic constriction (TAC) surgery. a, Hemodynamic quantification of maximum and minimum left ventricle volume (max of volume, V_{max}; min of volume, V_{min}), end-systolic volume (ESV), and end-diastolic volume (EDV) (n=5-9). **b,** Heart weight (HW), heart weight/body weight (HW/BW) ratio, and heart weight/tibia length (HW/TL) ratio of mice (n=7-11). Data among 4 groups were compared by two-way ANOVA test followed by Tukey post hoc test. *, P<0.05; ***, P<0.001.

Figure S5

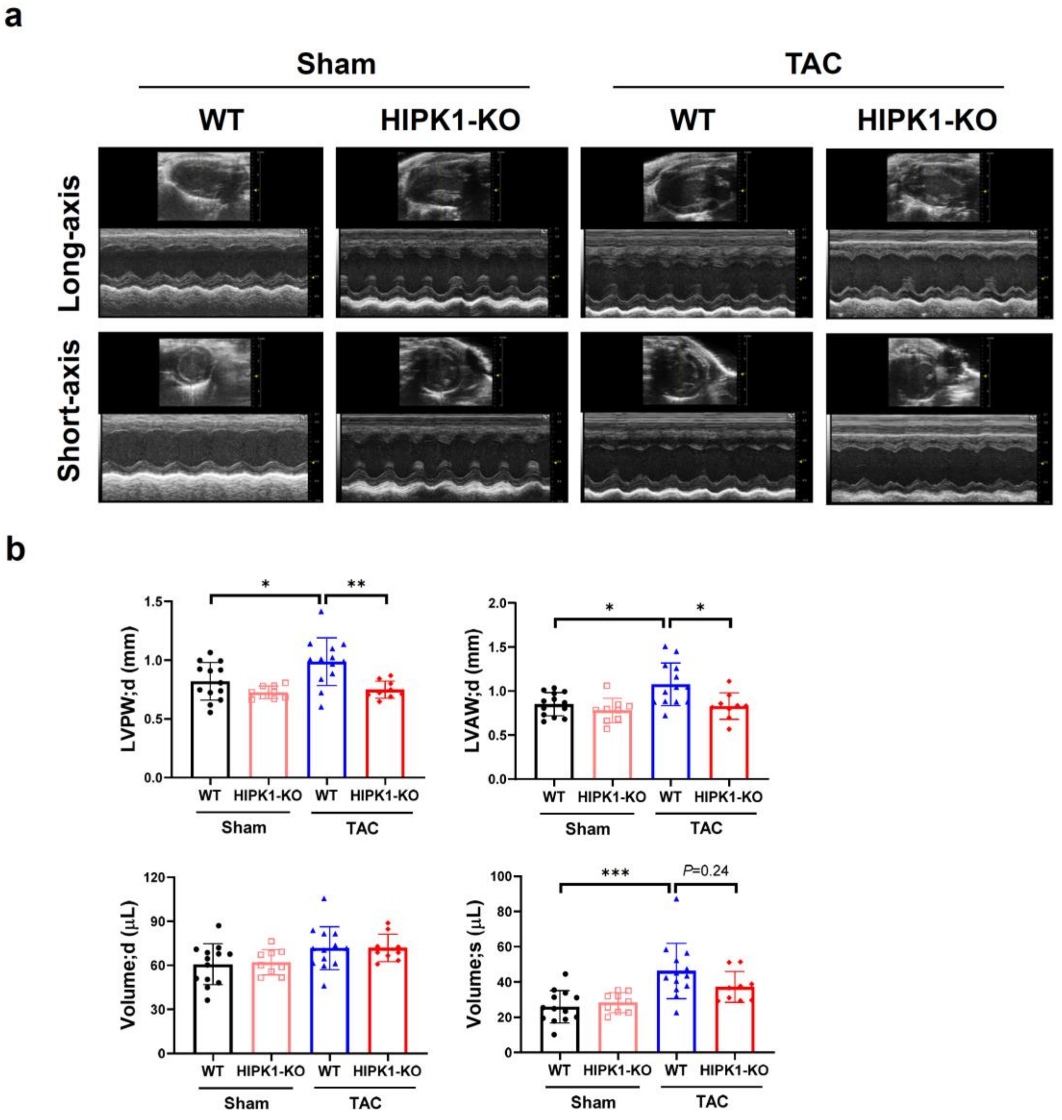

Figure S5. Echocardiography for left ventricle (LV) cardiac function of HIPK1 knockout (Line-1) mice at 8 weeks after transaortic constriction (TAC) surgery. a, Representative images showing the long-axis view and the corresponding short-axis view of left ventricle (LV) at the papillary muscle level. **b,** Statistical analysis of end-diastolic LV posterior wall thickness (LVPW;d) and anterior wall thickness (LVAW;d), and end-diastolic LV volume (Volume;d) and end-systolic LV volume (Volume;s) according to measurements of three consecutive cardiac cycles from the long-axis view of LV at the papillary muscle level (n=13 for WT mice, n=9 for HIPK1 KO mice). Data among 4 groups were compared by two-way ANOVA test followed by Tukey post hoc test. *, $P<0.05$; **, $P<0.01$; ***, $P<0.001$.

Figure S6

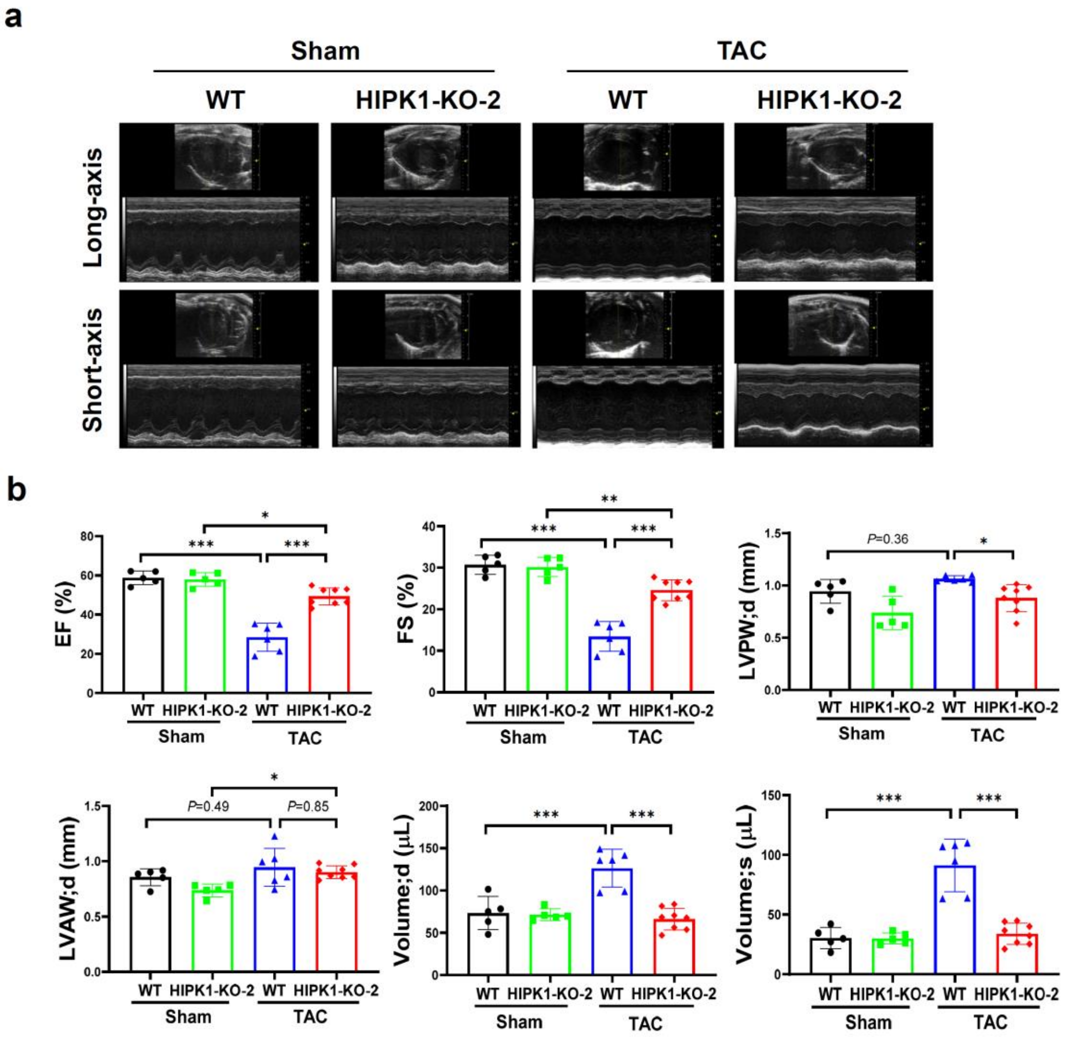

Figure S6. Echocardiography for left ventricle (LV) cardiac function of HIPK1 knockout (Line-2) mice at 6 weeks after transaortic constriction (TAC) surgery. a, Representative images showing the long-axis view and the corresponding short-axis view of left ventricle (LV) at the papillary muscle level. **b,** Statistical analysis of LV ejection fraction (EF) and fractional shortening (FS), end-diastolic LV posterior wall thickness (LVPW;d) and anterior wall thickness (LVAW;d), and end-diastolic LV volume (Volume;d) and end-systolic LV volume (Volume;s) according to measurements of three consecutive cardiac cycles from the long-axis view of LV at the papillary muscle level (n=5-8). Data among 4 groups were compared by two-way ANOVA test followed by Tukey post hoc test. *, $P<0.05$; **, $P<0.01$; ***, $P<0.001$.

Figure S7

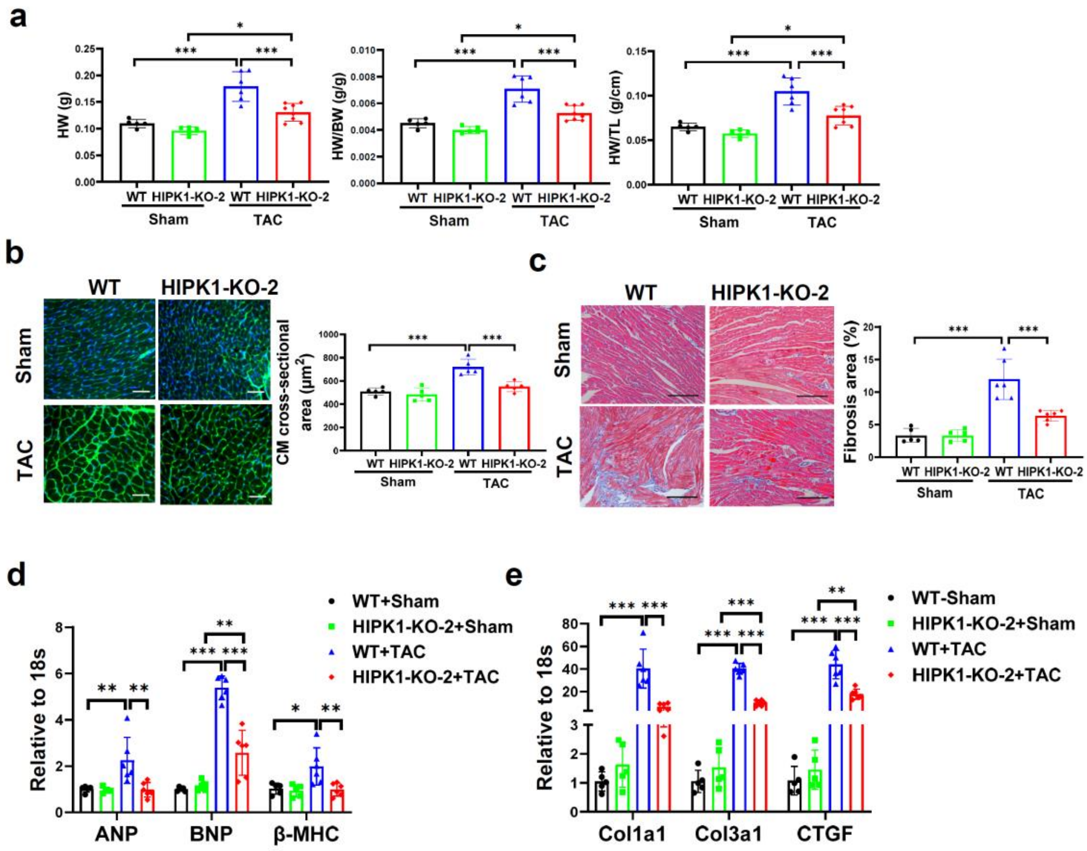

Figure S7. HIPK1 knockout mice (Line-2) are resistant to transaortic constriction (TAC)-induced pathological cardiac hypertrophy. a, Heart weight (HW), heart weight/body weight (HW/BW) ratio, and heart weight/tibia length (HW/TL) ratio of mice (n=5-8). **b,** Wheat germ agglutinin (WGA) staining for cardiomyocyte (CM) cross-sectional area (n=5). Scale bar=50 µm. **c,** Masson staining for cardiac fibrosis area (n=5-6). Scale bar=100 µm. **d and e,** qRT-PCR for ANP, BNP, and β-MHC **(d)** and Col1a1, Col3a1, and CTGF **(e)** in mice heart tissues after sham or TAC surgery (n=5-6). Data among 4 groups were compared by two-way ANOVA test followed by Tukey post hoc test. *, *P*<0.05; **, *P*<0.01; ***, *P*<0.001.

Figure S8

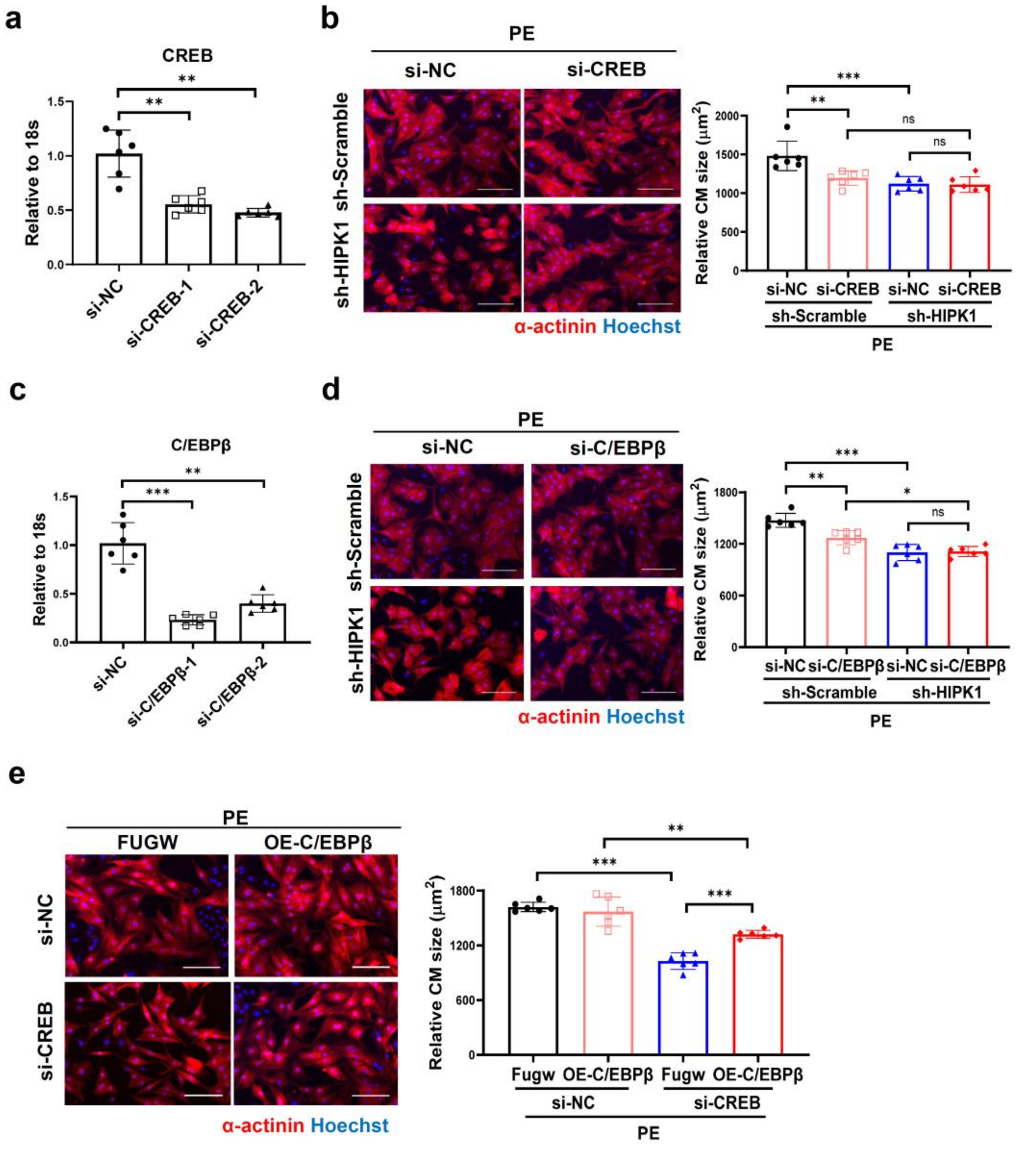

Figure S8. Function-rescue experiments between HIPK1 and CREB-C/EBPβ axis in neonatal rat cardiomyocytes (NRCMs) under phenylephrine (PE) stress. a and c, qRT-PCR for CREB **(a)** and C/EBPβ **(c)** in NRCMs transfected with CREB siRNA, C/EBPβ siRNA, or negative control, respectively (n=6). **b and d,** Immunofluorescent staining for α-actinin in NRCMs treated with sh-HIPK1 lentivirus and CREB siRNA **(b)** or C/EBPβ siRNA **(d)** (n=6). Scale bar=100 μm. **e,** Immunofluorescent staining for α-actinin in NRCMs treated with CREB siRNA and OE-C/EBPβ plasmid (n=6). Scale bar=100 μm. Data among 3 groups were compared by one-way ANOVA test. Data among 4 groups were compared by two-way ANOVA test followed by Tukey post hoc test. *, P<0.05; **, P<0.01; ***, P<0.001; ns, not significant.

Figure S9

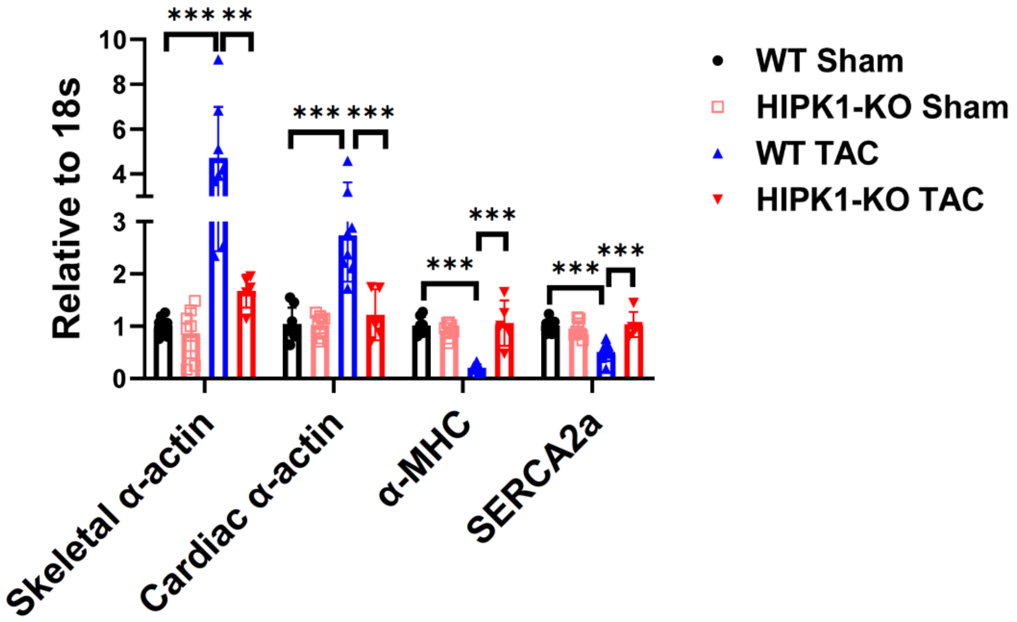

Figure S9. Suppressing HIPK1 reverses the genetic program changes associated with pathological cardiac hypertrophy. qRT-PCR for skeletal α-actin, cardiac α-actin, α-MHC, and SERCA2a in heart tissues from WT or HIPK1 knockout mice after sham or transaortic constriction (TAC) surgery (n=5-8). Data among 4 groups were compared by two-way ANOVA test followed by Tukey post hoc test. **, P<0.01; ***, P<0.001.

Figure S10

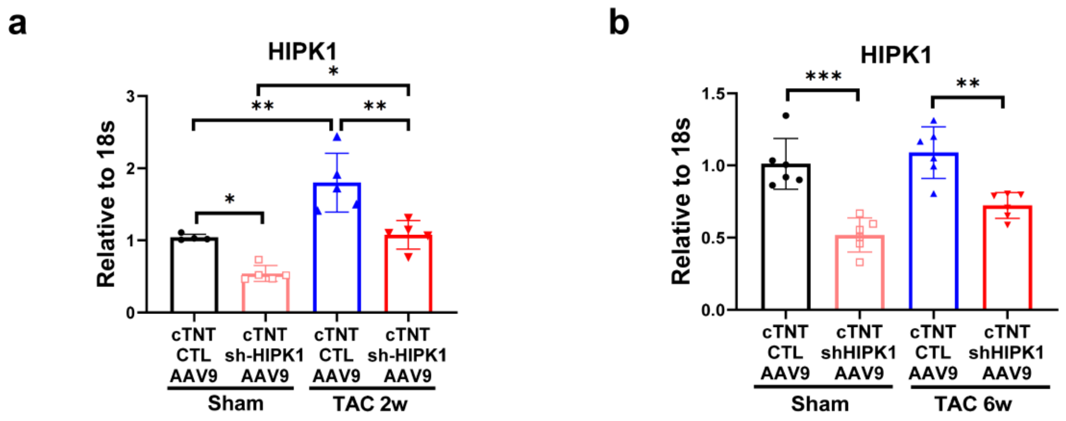

Figure S10. cTnT-shHIPK1-AAV9 injections significantly reduce HIPK1 level in mice hearts. Mice were treated via tail vein injections of cTnT promoter-mediated knockdown of HIPK1 AAV9 (cTnT-shHIPK1-AAV9) or control AAV9 vectors (cTnT-CTL-AAV9) and subsequently followed 1 week later by transaortic constriction (TAC) surgery to induce pathological cardiac hypertrophy. **a and b,** qRT-PCR was used to determine HIPK1 mRNA level in mice heart tissues at 2 weeks **(a**, n=4-5**)** and 6 weeks **(b**, n=6**)** after sham or TAC surgery, respectively. Data among 4 groups were compared by two-way ANOVA test followed by Tukey post hoc test. *, *P*<0.05; **, *P*<0.01; ***, *P*<0.001.

Figure S11

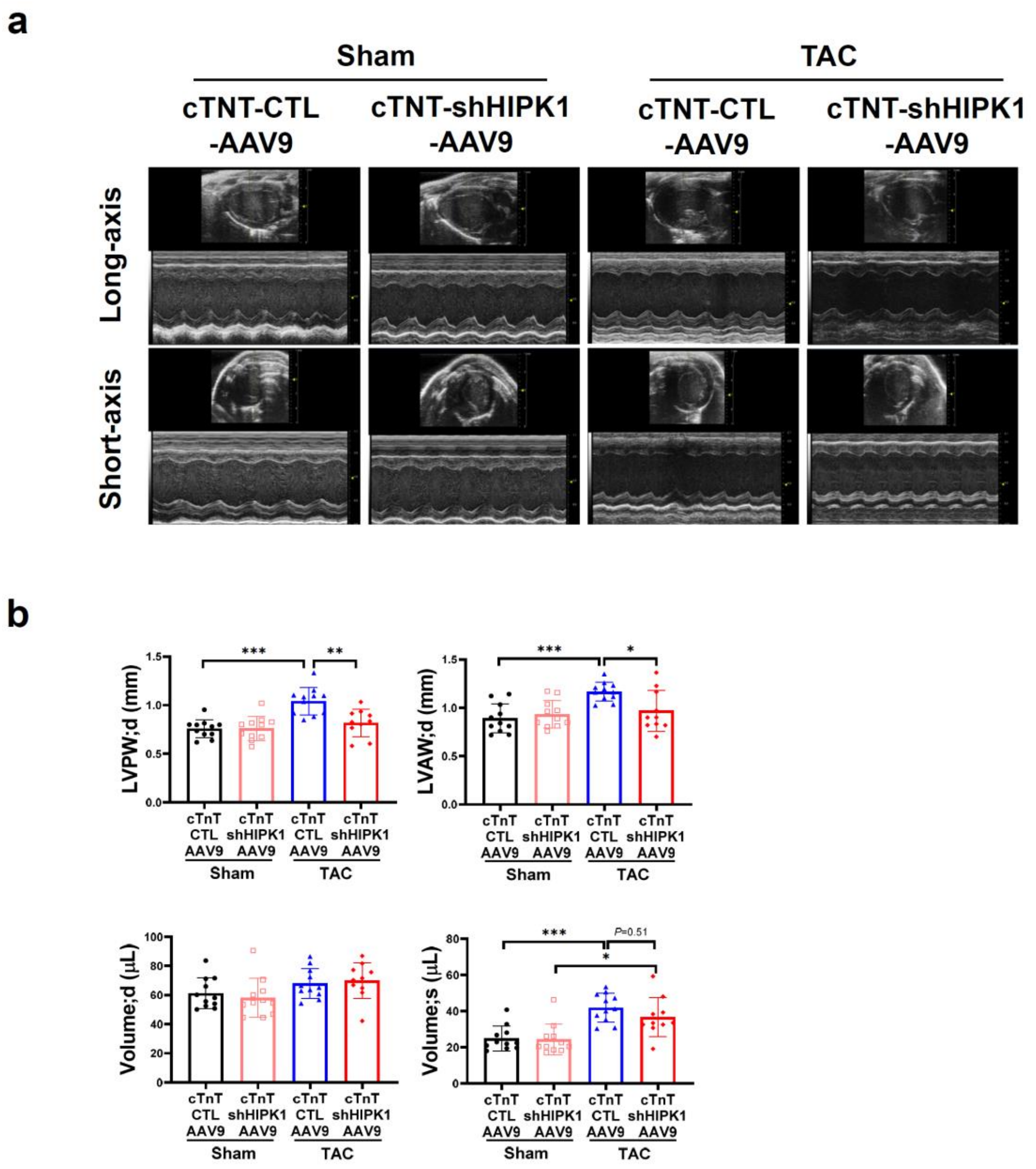

Figure S11. Echocardiography for left ventricle (LV) cardiac function of cTnT-shHIPK1-AAV9 injected mice at 6 weeks after transaortic constriction (TAC) surgery. a, Representative images showing the long-axis view and the corresponding short-axis view of left ventricle (LV) at the papillary muscle level. **b,** Statistical analysis of end-diastolic LV posterior wall thickness (LVPW;d) and anterior wall thickness (LVAW;d), and end-diastolic LV volume (Volume;d) and end-systolic LV volume (Volume;s) according to measurements of three consecutive cardiac cycles from the long-axis view of LV at the papillary muscle level (n=10-11). Data among 4 groups were compared by two-way ANOVA test followed by Tukey post hoc test. *, $P<0.05$; **, $P<0.01$; ***, $P<0.001$.

Figure S12

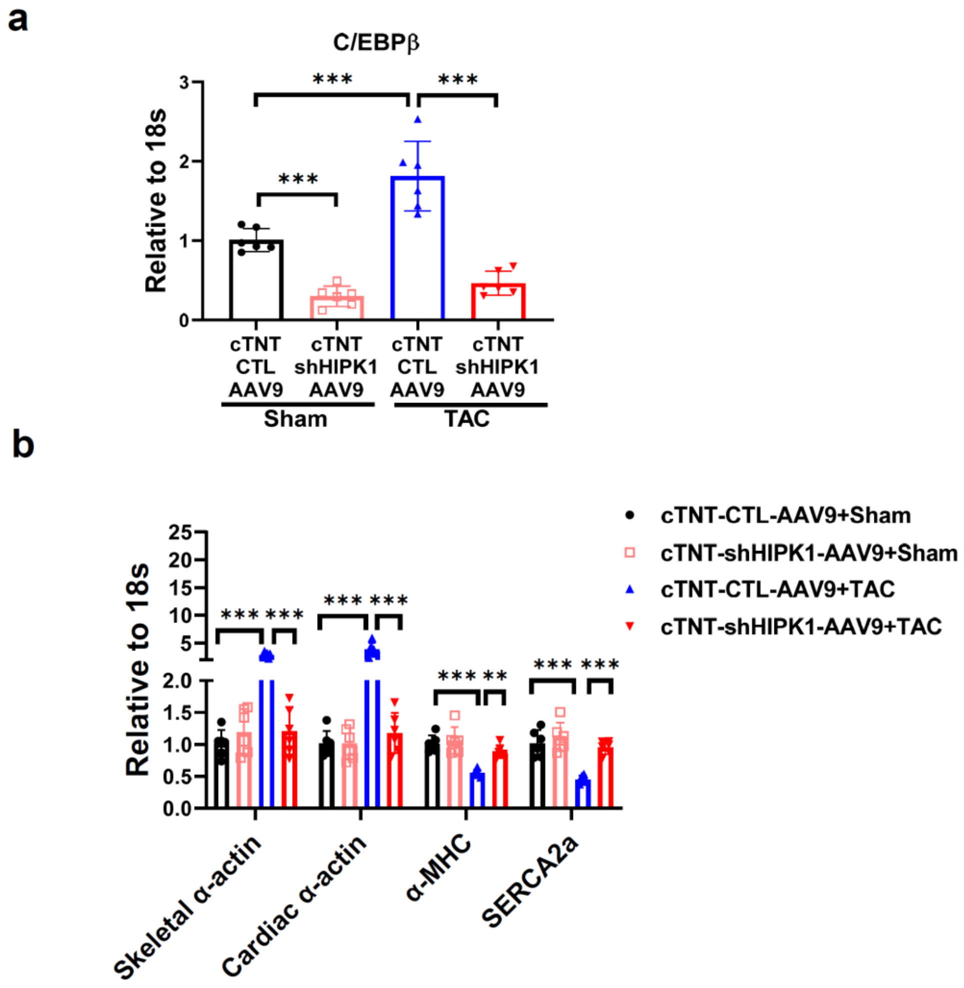

Figure S12. Knockdown of HIPK1 reduces C/EBPβ and its downstream hypertrophic response genes in transaortic constriction (TAC)-induced pathological cardiac hypertrophy. a and b, qRT-PCR for C/EBPβ **(a)** and skeletal α-actin, cardiac α-actin, α-MHC, and SERCA2a **(b)** in heart tissues from sham- or TAC-operated mice injected with cTnT promoter-mediated knockdown of HIPK1 AAV9 (cTnT-shHIPK1-AAV9) or control AAV9 vectors (cTnT-CTL-AAV9) (n=6). Data among 4 groups were compared by two-way ANOVA test followed by Tukey post hoc test. **, *P*<0.01; ***, *P*<0.001.

Figure S13

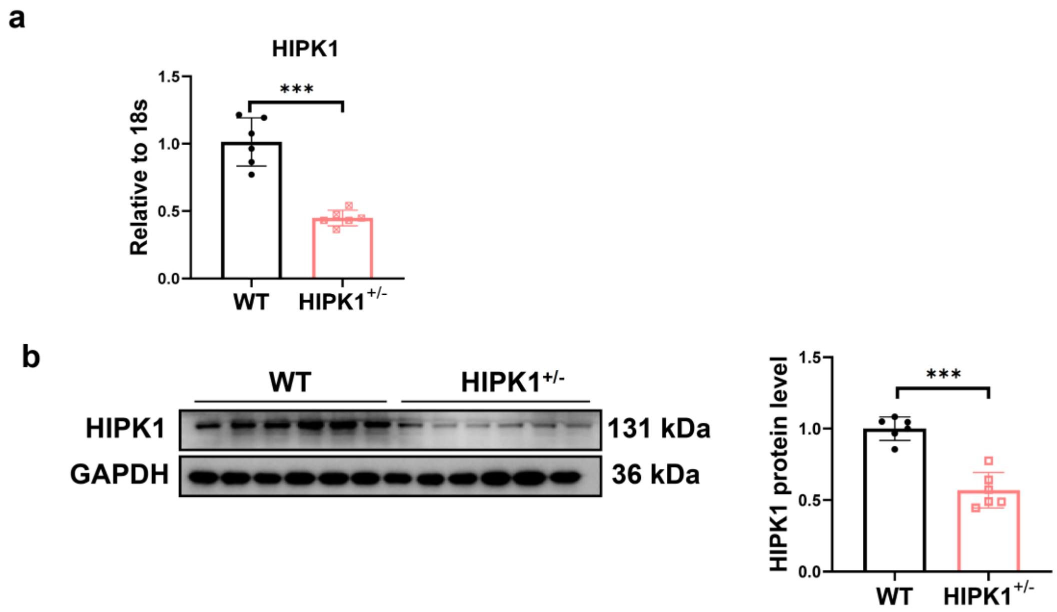

Figure S13. Mice with hetero-knockout of HIPK1 (HIPK1$^{+/-}$) have reduced HIPK1 expression in heart tissues. a and b, qRT-PCR **(a)** and Western blot **(b)** for HIPK1 in heart tissues from HIPK1$^{+/-}$ mice and wild type (WT) mice (n=6). Data between 2 groups were compared by independent-sample two-tailed Student's t-test. ***, *P*<0.001.

Figure S14

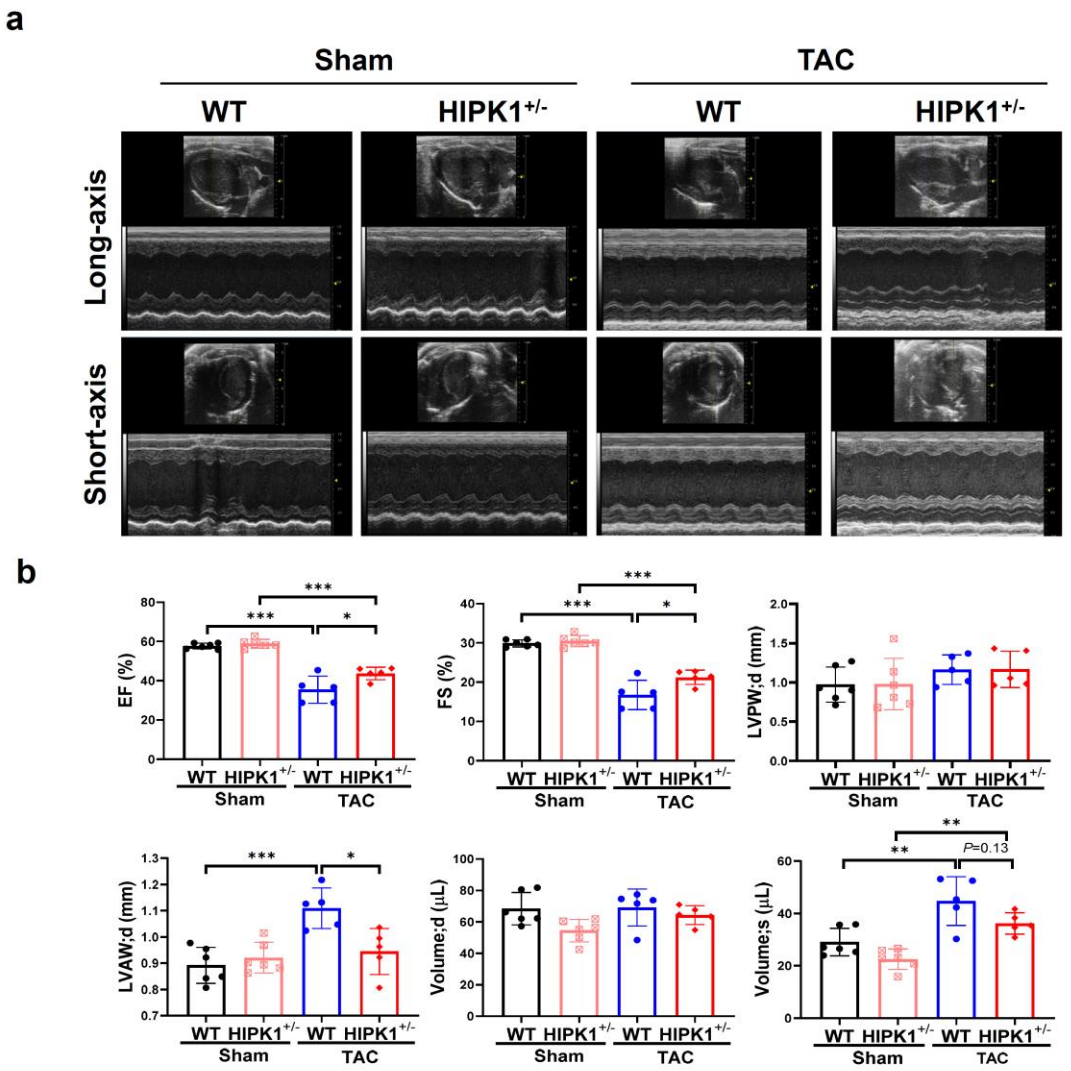

Figure S14. HIPK1 hetero-knockout mice have improved cardiac function at 8 weeks after transaortic constriction (TAC) surgery. a, Echocardiography for left ventricle (LV) systolic function of mice with representative images showing the long-axis view and the corresponding short-axis view of LV at the papillary muscle level. **b,** Statistical analysis of LV ejection fraction (EF) and fractional shortening (FS), end-diastolic LV posterior wall thickness (LVPW;d) and anterior wall thickness (LVAW;d), and end-diastolic LV volume (Volume;d) and end-systolic LV volume (Volume;s) according to measurements of three consecutive cardiac cycles from the long-axis view of LV at the papillary muscle level (n=5-6). Data among 4 groups were compared by two-way ANOVA test followed by Tukey post hoc test. *, $P<0.05$; **, $P<0.01$; ***, $P<0.001$.

Figure S15

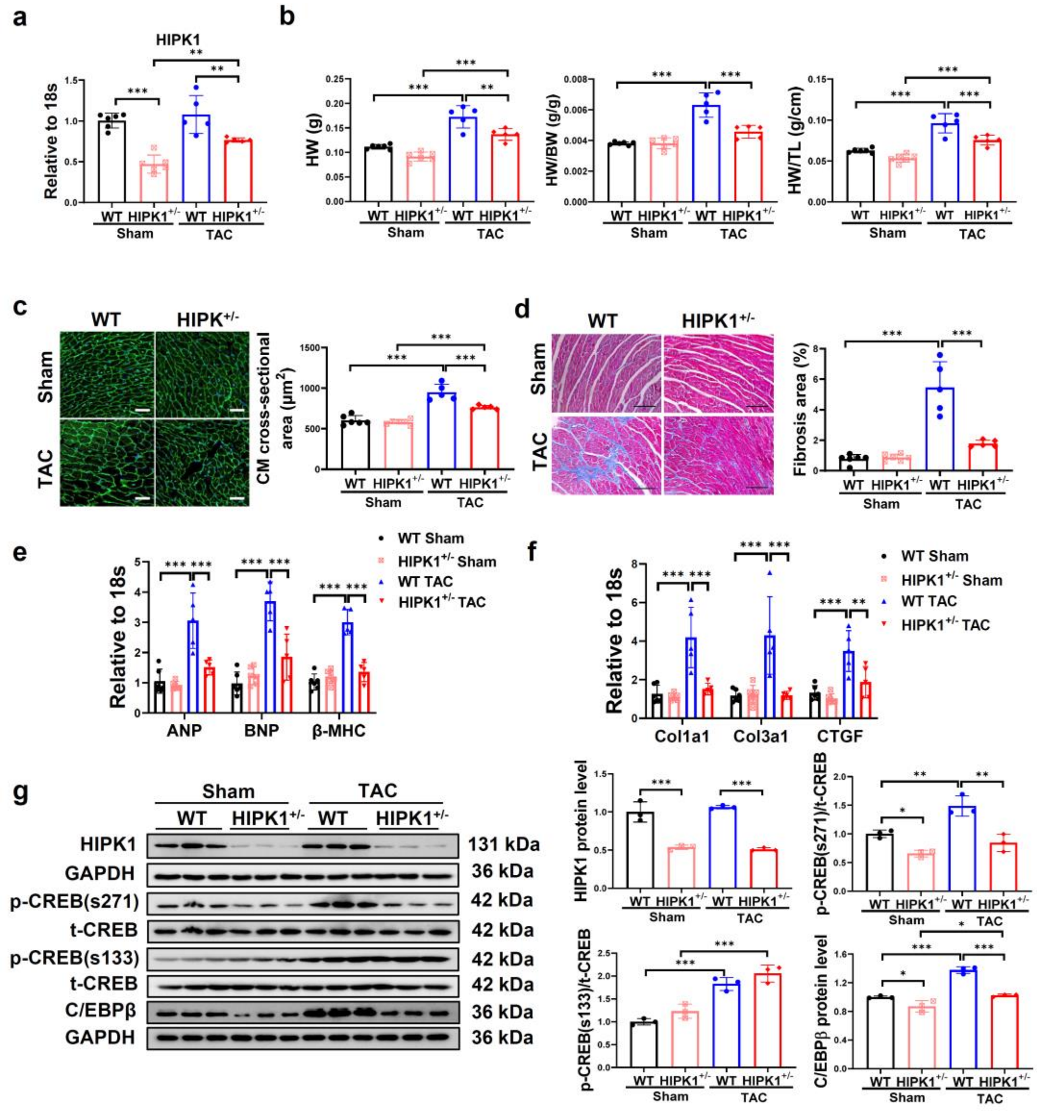

Figure S15. HIPK1 hetero-knockout is sufficient to attenuate transaortic constriction (TAC)-induced pathological cardiac hypertrophy. a, qRT-PCR for HIPK1 in heart tissues from sham-operated mice or mice at 8 weeks post TAC surgery (n=5-6). **b,** Heart weight (HW), heart weight/body weight (HW/BW) ratio, and heart weight/tibia length (HW/TL) ratio of mice (n=5-6). **c,** Wheat germ agglutinin (WGA) staining for cardiomyocyte (CM) cross-sectional area (n=5-6). Scale bar=50 μm. **d,** Masson staining for cardiac fibrosis area (n=5-6). Scale bar=100 μm. **e and f,** qRT-PCR for ANP, BNP, and β-MHC **(e)** and Col1a1, Col3a1, and CTGF **(f)** in mice heart tissues after sham or TAC surgery (n=5-6). **g,** Western blot for HIPK1, CREB phosphorylation, and C/EBPβ expressions in mice heart tissues after sham or TAC surgery (n=3). Data among 4 groups were compared by two-way ANOVA test followed by Tukey post hoc test. *, *P*<0.05; **, *P*<0.01; ***, *P*<0.001.

Figure S16

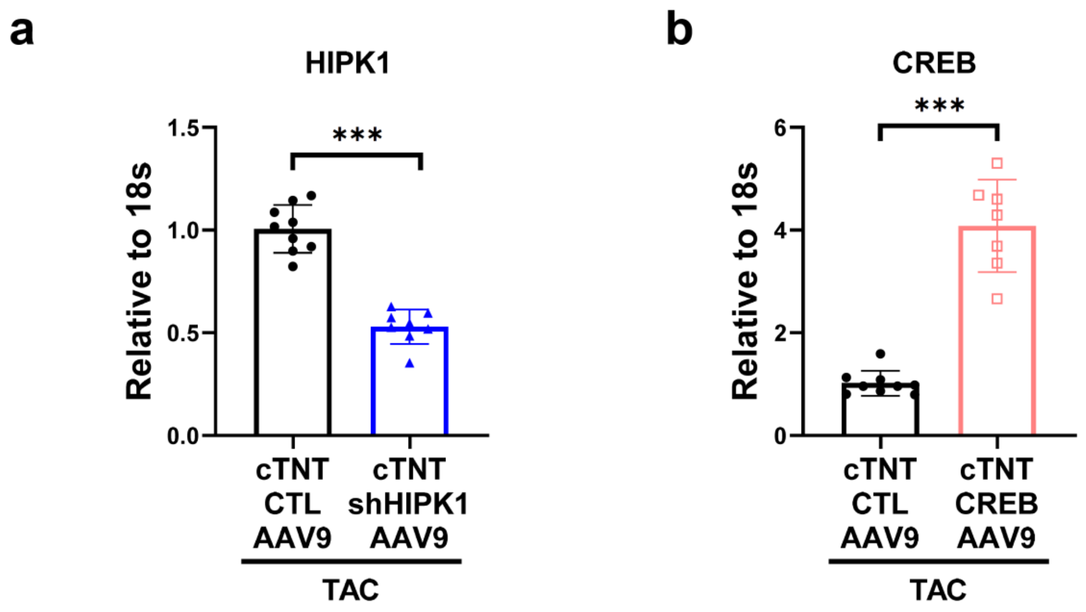

Figure S16. Validation of AAV9-mediated gene expression changes in mice hearts. Mice were treated via tail vein injections of cTnT promoter-mediated knockdown of HIPK1 AAV9 (cTnT-shHIPK1-AAV9), cTnT promoter-mediated overexpression of CREB AAV9 (cTnT-CREB-AAV9), or control AAV9 vectors (cTnT-CTL-AAV9) and subsequently followed 1 week later by transaortic constriction (TAC) surgery to induce pathological cardiac hypertrophy. **a and b**, at 6 weeks post TAC, qRT-PCR was used to determine HIPK1 **(a)** and CREB **(b)** mRNA levels in mice heart tissues (n=7-9). Data between 2 groups were compared by independent-sample two-tailed Student's t-test. ***, $P<0.001$.

Figure S17

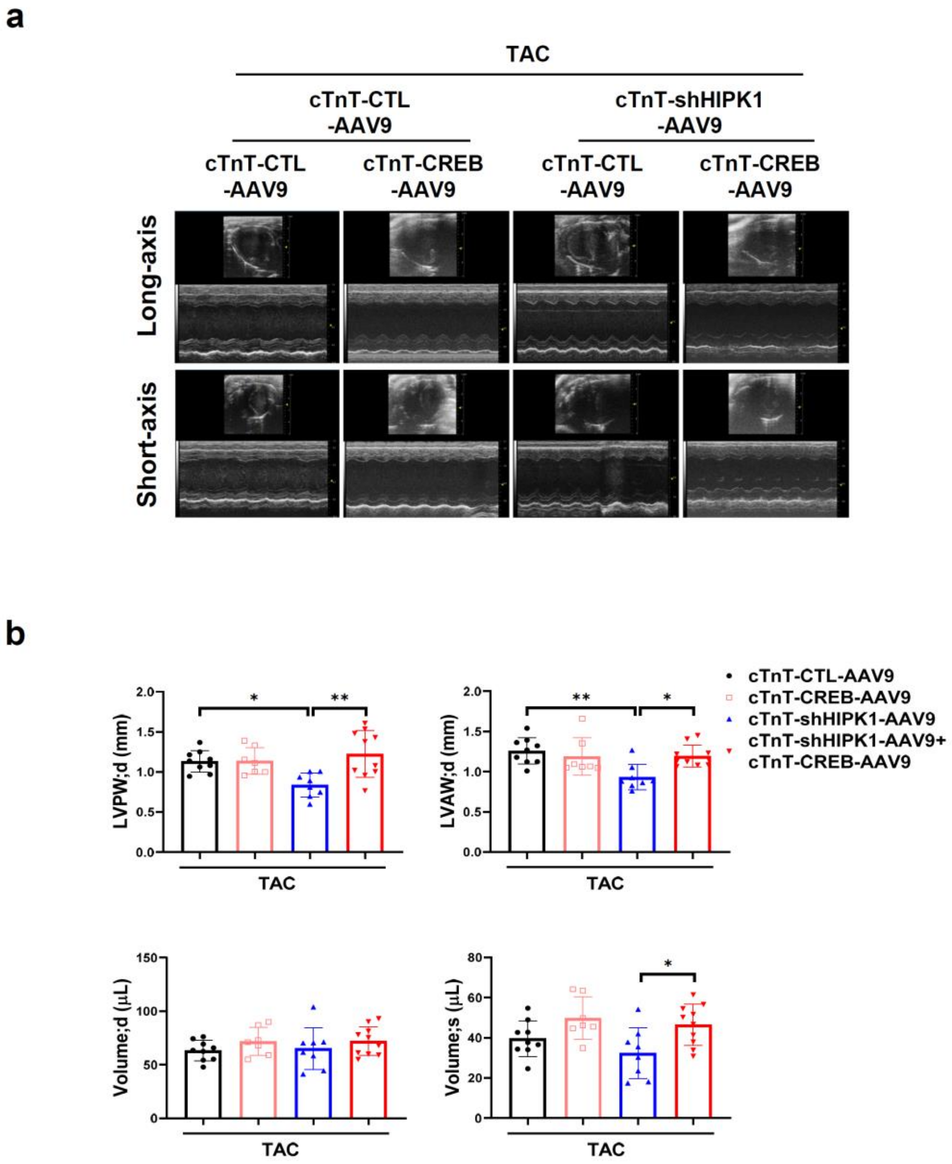

Figure S17. Echocardiography for left ventricle (LV) cardiac function of mice with cTnT-shHIPK1-AAV9 and/or cTnT-CREB-AAV9 injections at 6 weeks after transaortic constriction (TAC) surgery. a, Representative images showing the long-axis view and the corresponding short-axis view of left ventricle (LV) at the papillary muscle level. **b,** Statistical analysis of end-diastolic LV posterior wall thickness (LVPW;d) and anterior wall thickness (LVAW;d), and end-diastolic LV volume (Volume;d) and end-systolic LV volume (Volume;s) according to measurements of three consecutive cardiac cycles from the long-axis view of LV at the papillary muscle level (n=7-10). Data among 4 groups were compared by two-way ANOVA test followed by Tukey post hoc test. *, $P<0.05$; **, $P<0.01$; ***, $P<0.001$.

Figure S18

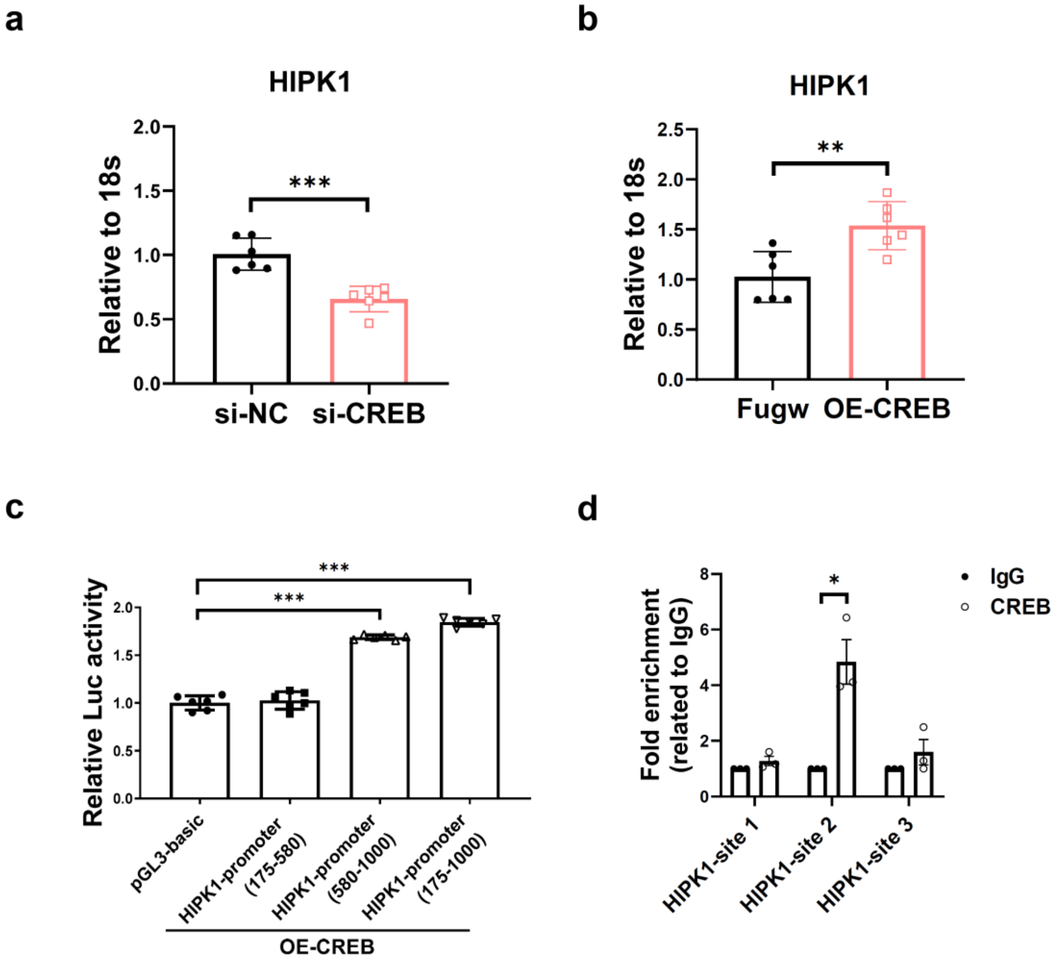

Figure S18. CREB positively regulates HIPK1 in cardiomyocytes. a and b, qRT-PCR for HIPK1 in neonatal rat cardiomyocytes (NRCM) transfected with CREB siRNA (si-CREB) and negative control (si-NC) (**a**, n=6), or with OE-CREB plasmid and Fugw control vector (**b**, n=6). **c,** Luciferase reporter assay for detection of the regulation between CREB and HIPK1 (n=6). **d,** ChIP-PCR assay for detection of the enrichment of CREB to the promoter region of HIPK1 (n=3). Data between 2 groups were compared by independent-sample two-tailed Student's t-test. Data among 4 groups were compared by one-way ANOVA test. *, P<0.05; **, P<0.01; ***, P<0.001.

Figure S19

a **HIPK1 KO-Founder-1:**

Wild type: CAATCTTCCTGCTTACGACCAGGGCCTCCTTCTCCCAGCTCC

Mutant: CAATCTTCCTGC-------------CTCCTTCTCCCAGCTCC

b **HIPK1 KO-Founder-2:**

Wild type: CAATCTTCCTGCTTACG-ACCAGGGCCTCCTTCT

Mutant: CAATCTTCCTGCTTACGGACCAGGGCCTCCTTCT

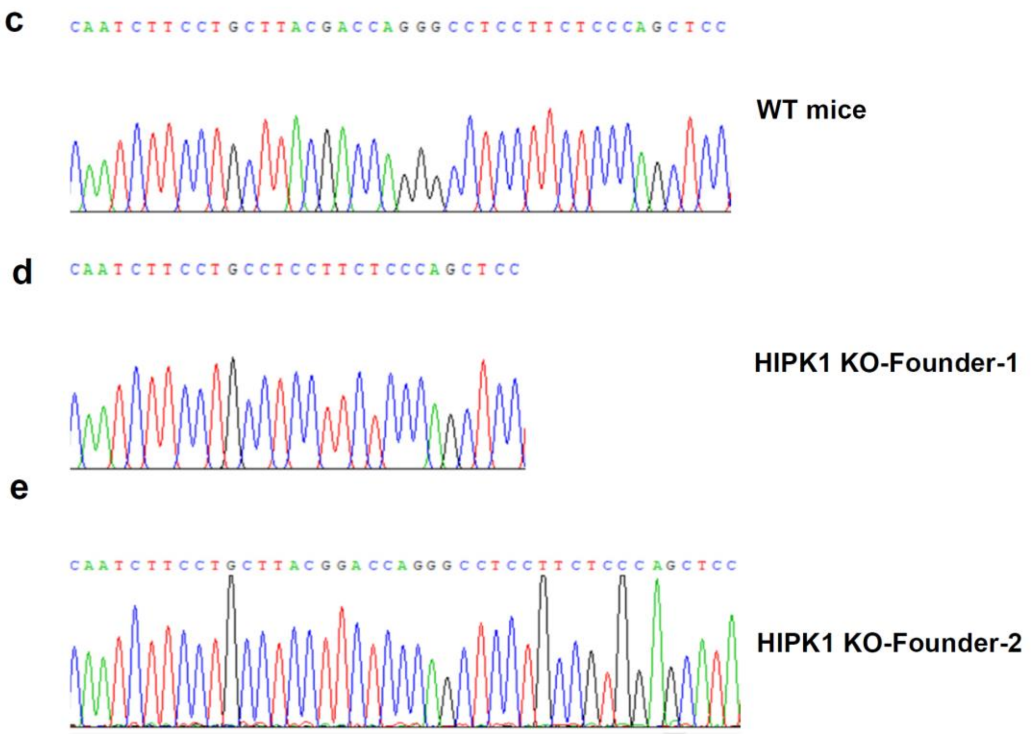

Figure S19. Generation and DNA sequences of HIPK1 knockout (KO) mice. a, HIPK1 KO mice (Founder-1) was generated with 13 bp deletion before CTCCTTCTCCCAGCTCC in HIPK1 genomic DNA. **b,** HIPK1 KO mice (Founder-2) was generated with one G insertion before ACCAGGGCCTCCTTCT in HIPK1 genomic DNA. **c-e,** Genotypes were confirmed by DNA sequencing of tail DNA as demonstrated for wild type (WT) mice **(c)**, Founder-1 **(d)** and Founder-2 **(e)**, respectively.

Supplemental Tables

Table S1. Echocardiography parameters of wild type (WT) mice *versus* HIPK1 knockout (KO) mice (Line-1) at 4 weeks after TAC surgery.

	WT+Sham (n=7)	HIPK1 KO+Sham (n=8)	WT+TAC 4w (n=10)	HIPK1 KO+TAC 4w (n=11)
**Heart rate (bpm)**	470.20±41.25	487.30±41.93	474.20±50.57	478.90±33.39
**EF (%)**	60.25±8.05	60.63±6.16	31.19±6.51***	54.63±5.58$^{\#\#\#}$
**FS (%)**	31.74±5.23	31.81±4.20	14.54±3.34***	27.86±3.50$^{\#\#\#}$
**LV Mass (mg)**	118.5±29.2	101.5±22.6	211.7±44.7***	135.3±15.9$^{\#\#\#}$
**LVPW;s (mm)**	1.26±0.26	1.18±0.16	1.35±0.17	1.31±0.14
**LVPW;d (mm)**	0.88±0.19	0.80±0.14	1.19±0.20**	0.99±0.11$^{\#}$
**LVAW;s (mm)**	1.30±0.17	1.27±0.18	1.42±0.11	1.34±0.09
**LVAW;d (mm)**	0.88±0.18	0.87±0.16	1.20±0.13***	0.94±0.11$^{\#\#}$
**Diameter;s (mm)**	2.53±0.31	2.41±0.38	3.45±0.47***	2.69±0.29$^{\#\#\#}$
**Diameter;d (mm)**	3.70±0.34	3.51±0.35	4.03±0.44	3.72±0.27
**Volume;s (µL)**	23.42±6.69	21.14±7.80	50.56±16.81***	27.15±7.39$^{\#\#\#}$
**Volume;d (µL)**	58.71±12.53	52.10±12.03	72.55±19.12	59.21±10.51

BPM, beat per minute; EF, ejection fraction; FS, fractional shortening; LV, left ventricle; LVPW;s, end-systolic LV posterior wall thickness; LVPW;d, end-diastolic LV posterior wall thickness; LVAW;s, end-systolic LV anterior wall thickness; LVAW;d, end-diastolic LV anterior wall

thickness; Diameter;s, end-systolic LV diameter; Diameter;d, end-diastolic LV diameter; Volume;s, end-systolic LV volume; Volume;d, end-diastolic LV volume. Data are presented as mean±SD. Data among 4 groups were compared by two-way ANOVA test followed by Tukey post hoc test. **, *P*<0.01 compared to corresponding Sham group; ***, *P*<0.001 compared to corresponding Sham group; ##, *P*<0.01 compared to WT+TAC group; ###, *P*<0.001 compared to WT+TAC group.

Table S2. Echocardiography parameters of wild type (WT) mice *versus* HIPK1 knockout (KO) mice (Line-1) at 8 weeks after TAC surgery.

	**WT+Sham (n=13)**	**HIPK1 KO+Sham (n=9)**	**WT+TAC (n=13)**	**HIPK1 KO+TAC (n=9)**
**Heart rate (bpm)**	498.20±40.52	458.00±43.84	488.80±40.54	447.40±43.11
**EF (%)**	58.44±6.46	54.62±5.76	36.83±8.98***	48.79±5.86$^{##}$
**FS (%)**	30.46±4.31	27.91±3.76	17.59±4.74***	24.38±3.49$^{##}$
**LV Mass (mg)**	110.9±14.3	99.8±17.6	168.2±30.5***	118.0±22.1$^{###}$
**LVPW;s (mm)**	1.12±0.20	1.01±0.15	1.15±0.25	0.99±0.09
**LVPW;d (mm)**	0.82±0.16	0.73±0.05	0.99±0.20*	0.75±0.07$^{##}$
**LVAW;s (mm)**	1.29±0.20	1.19±0.17	1.36±0.30	1.16±0.19
**LVAW;d (mm)**	0.85±0.13	0.78±0.14	1.08±0.24*	0.83±0.15$^{#}$
**Diameter;s (mm)**	2.62±0.39	2.74±0.23	3.33±0.45***	3.06±0.29
**Diameter;d (mm)**	3.75±0.37	3.80±0.22	4.03±0.34	4.04±0.22
**Volume;s (μL)**	25.89±9.14	28.28±5.69	46.28±15.71***	37.21±8.78
**Volume;d (μL)**	60.75±13.88	62.15±8.59	71.76±14.59	71.94±9.34

BPM, beat per minute; EF, ejection fraction; FS, fractional shortening; LV, left ventricle; LVPW;s, end-systolic LV posterior wall thickness; LVPW;d, end-diastolic LV posterior wall thickness; LVAW;s, end-systolic LV anterior wall thickness; LVAW;d, end-diastolic LV anterior wall

thickness; Diameter;s, end-systolic LV diameter; Diameter;d, end-diastolic LV diameter; Volume;s, end-systolic LV volume; Volume;d, end-diastolic LV volume. Data are presented as mean±SD. Data among 4 groups were compared by two-way ANOVA test followed by Tukey post hoc test. *, *P*<0.05 compared to corresponding Sham group; ***, *P*<0.001 compared to corresponding Sham group; #, *P*<0.05 compared to WT+TAC group; ##, *P*<0.01 compared to WT+TAC group; ###, *P*<0.001 compared to WT+TAC group.

Table S3. Echocardiography parameters of wild type (WT) mice *versus* HIPK1 knockout (KO) mice (Line-2) at 6 weeks after TAC surgery.

	WT+Sham (n=5)	HIPK1-KO-2+Sham (n=5)	WT+TAC (n=6)	HIPK1-KO-2+TAC (n=8)
**Heart rate (bpm)**	461.5±32.8	448.3±24.7	455.6±34.7	461.3±21.6
**EF (%)**	58.73±3.44	57.91±3.51	28.45±7.13***	49.32±4.32$^{*,\#\#\#}$
**FS (%)**	30.71±2.28	30.14±2.30	13.42±3.58***	24.58±2.49$^{**,\#\#\#}$
**LV Mass (mg)**	141.2±22.1	106.7±10.9	240.2±21.2***	131.2±24.7$^{\#\#\#}$
**LVPW;s (mm)**	1.24±0.04	1.16±0.18	1.24±0.05	1.10±0.12
**LVPW;d (mm)**	0.94±0.11	0.74±0.16	1.06±0.03	0.88±0.13$^{\#}$
**LVAW;s (mm)**	1.35±0.16	1.18±0.08	1.18±0.22	1.27±0.06
**LVAW;d (mm)**	0.85±0.08	0.74±0.06	0.95±0.17	0.90±0.06$^{\#}$
**Diameter;s (mm)**	2.81±0.34	2.82±0.18	4.45±0.48***	2.94±0.33$^{\#\#\#}$
**Diameter;d (mm)**	4.05±0.47	4.03±0.16	5.13±0.41***	3.89±0.33$^{\#\#\#}$
**Volume;s (µL)**	30.35±8.81	30.10±4.71	91.20±21.98***	33.89±8.96$^{\#\#\#}$
**Volume;d (µL)**	73.23±19.68	71.27±6.92	126.30±22.39***	66.09±13.08$^{\#\#\#}$

BPM, beat per minute; EF, ejection fraction; FS, fractional shortening; LV, left ventricle; LVPW;s, end-systolic LV posterior wall thickness; LVPW;d, end-diastolic LV posterior wall thickness; LVAW;s, end-systolic LV anterior wall thickness; LVAW;d, end-diastolic LV anterior wall

thickness; Diameter;s, end-systolic LV diameter; Diameter;d, end-diastolic LV diameter; Volume;s, end-systolic LV volume; Volume;d, end-diastolic LV volume. Data are presented as mean±SD. Data among 4 groups were compared by two-way ANOVA test followed by Tukey post hoc test. *, $P<0.05$ compared to corresponding Sham group; **, $P<0.01$ compared to corresponding Sham group; ***, $P<0.001$ compared to corresponding Sham group; #, $P<0.05$ compared to WT+TAC group; ###, $P<0.001$ compared to WT+TAC group.

Table S4. Echocardiography parameters of mice injected with cTnT-shHIPK1-AAV9 *versus* control AAV9 at 6 weeks after TAC surgery.

	cTnT-CTL-AAV9+Sham (n=11)	cTnT-shHIPK1-AAV9+Sham (n=11)	cTnT-CTL-AAV9+TAC (n=11)	cTnT-shHIPK1-AAV9+TAC (n=10)
**Heart rate (bpm)**	504.6±43.2	508.9±37.9	497.3±46.3	492.4±45.6
**EF (%)**	59.82±3.92	58.69±4.72	38.50±5.46***	48.07±8.35***,##
**FS (%)**	31.31±2.52	30.5±3.07	18.38±2.90***	23.99±4.72***,##
**LV Mass (mg)**	111.6±20.9	111.5±20.8	181.4±26.8***	136.9±30.5##
**LVPW;s (mm)**	1.12±0.13	1.13±0.13	1.30±0.17*	1.07±0.18##
**LVPW;d (mm)**	0.76±0.09	0.76±0.12	1.04±0.14***	0.82±0.14##
**LVAW;s (mm)**	1.31±0.16	1.36±0.13	1.39±0.13	1.31±0.30
**LVAW;d (mm)**	0.89±0.15	0.93±0.14	1.17±0.10***	0.97±0.21#
**Diameter;s (mm)**	2.59±0.27	2.56±0.33	3.22±0.26***	3.03±0.36**
**Diameter;d (mm)**	3.77±0.27	3.68±0.34	3.94±0.25	3.98±0.32
**Volume;s (µL)**	24.92±6.81	24.41±8.48	41.92±7.91***	36.72±10.77*
**Volume;d (µL)**	61.23±10.65	58.13±13.35	67.93±10.29	69.89±12.41

BPM, beat per minute; EF, ejection fraction; FS, fractional shortening; LV, left ventricle; LVPW;s, end-systolic LV posterior wall thickness; LVPW;d, end-diastolic LV posterior wall thickness; LVAW;s, end-systolic LV anterior wall thickness; LVAW;d, end-diastolic LV anterior wall

thickness; Diameter;s, end-systolic LV diameter; Diameter;d, end-diastolic LV diameter; Volume;s, end-systolic LV volume; Volume;d, end-diastolic LV volume. Data are presented as mean±SD. Data among 4 groups were compared by two-way ANOVA test followed by Tukey post hoc test. *, *P*<0.05 compared to corresponding Sham group; **, *P*<0.01 compared to corresponding Sham group; ***, *P*<0.001 compared to corresponding Sham group; #, *P*<0.05 compared to WT+TAC group; ##, *P*<0.01 compared to WT+TAC group.

Table S5. Echocardiography parameters of wild type (WT) *versus* HIPK1 hetero-knockout mice at 8 weeks after TAC surgery.

	WT+Sham (n=6)	HIPK1$^{+/-}$+Sham (n=6)	WT+TAC (n=5)	HIPK1$^{+/-}$+TAC (n=5)
**Heart rate (bpm)**	505.9±20.2	504.9±29.3	477.2±53.3	508.1±14.6
**EF (%)**	57.65±1.50	58.86±2.24	35.43±6.93***	43.71±3.21***,#
**FS (%)**	29.88±0.89	30.47±1.42	16.76±3.74***	21.23±1.82***,#
**LV Mass (mg)**	143±27.3	125.9±21.7	192.8±43.2	163.9±27.0
**LVPW;s (mm)**	1.32±0.23	1.30±0.31	1.32±0.18	1.42±0.20
**LVPW;d (mm)**	0.97±0.22	0.98±0.33	1.16±0.19	1.17±0.23
**LVAW;s (mm)**	1.37±0.07	1.34±0.07	1.40±0.13	1.28±0.06
**LVAW;d (mm)**	0.89±0.07	0.92±0.06	1.11±0.08***	0.94±0.09#
**Diameter;s (mm)**	2.77±0.20	2.50±0.18	3.30±0.30**	3.04±0.14**
**Diameter;d (mm)**	3.95±0.25	3.59±0.20	3.97±0.30	3.86±0.15
**Volume;s (μL)**	29.06±5.28	22.51±3.90	44.74±9.31**	36.19±4.08**
**Volume;d (μL)**	68.38±10.34	54.45±7.17	69.22±11.77	64.29±6.04

BPM, beat per minute; EF, ejection fraction; FS, fractional shortening; LV, left ventricle; LVPW;s, end-systolic LV posterior wall thickness; LVPW;d, end-diastolic LV posterior wall thickness; LVAW;s, end-systolic LV anterior wall thickness; LVAW;d, end-diastolic LV anterior wall

thickness; Diameter;s, end-systolic LV diameter; Diameter;d, end-diastolic LV diameter; Volume;s, end-systolic LV volume; Volume;d, end-diastolic LV volume. Data are presented as mean±SD. Data among 4 groups were compared by two-way ANOVA test followed by Tukey post hoc test. **, *P*<0.01 compared to corresponding Sham group; ***, *P*<0.001 compared to corresponding Sham group; #, *P*<0.05 compared to WT+TAC group.

Table S6. Echocardiography parameters of mice injected with cTnT-shHIPK1-AAV9 and/or cTnT-CREB-AAV9 at 6 weeks after TAC surgery.

	cTnT-CTL-AAV9+TAC (n=9)	cTnT-CREB-AAV9+TAC (n=7)	cTnT-shHIPK1-AAV9+TAC (n=8)	cTnT-shHIPK1-AAV9+ cTnT-CREB-AAV9+TAC (n=10)
**Heart rate (bpm)**	490.9±32.3	506.1±34.1	492.8±23.0	492.4±30.5
**EF (%)**	37.85±6.00	30.84±4.33	51.5±6.77***	35.83±4.65$^{\#\#\#}$
**FS (%)**	17.98±3.19	14.32±2.20	25.97±4.06***	16.95±2.48$^{\#\#\#}$
**LV Mass (mg)**	195.1±35.7	201.9±34.8	128.9±38.2**	215.7±38.7$^{\#\#\#}$
**LVPW;s (mm)**	1.33±0.10	1.31±0.18	1.13±0.19	1.39±0.30
**LVPW;d (mm)**	1.13±0.13	1.14±0.17	0.84±0.15*	1.23±0.29$^{\#\#}$
**LVAW;s (mm)**	1.53±0.14	1.42±0.29	1.32±0.16	1.46±0.15
**LVAW;d (mm)**	1.26±0.16	1.19±0.23	0.93±0.16**	1.20±0.14$^{\#}$
**Diameter;s (mm)**	3.14±0.29	3.46±0.31	2.86±0.47	3.36±0.31$^{\#}$
**Diameter;d (mm)**	3.82±0.24	4.03±0.31	3.85±0.48	4.04±0.32
**Volume;s (μL)**	39.62±8.88	49.85±10.59	32.37±12.63	46.52±10.20$^{\#}$
**Volume;d (μL)**	63.19±9.46	71.84±13.08	65.20±19.47	72.13±13.49

BPM, beat per minute; EF, ejection fraction; FS, fractional shortening; LV, left ventricle; LVPW;s, end-systolic LV posterior wall thickness;

LVPW;d, end-diastolic LV posterior wall thickness; LVAW;s, end-systolic LV anterior wall thickness; LVAW;d, end-diastolic LV anterior wall thickness; Diameter;s, end-systolic LV diameter; Diameter;d, end-diastolic LV diameter; Volume;s, end-systolic LV volume; Volume;d, end-diastolic LV volume. Data are presented as mean±SD. Data among 4 groups were compared by two-way ANOVA test followed by Tukey post hoc test. *, $P<0.05$ compared to corresponding Sham group; **, $P<0.01$ compared to corresponding Sham group; ***, $P<0.001$ compared to corresponding Sham group; #, $P<0.05$ compared to WT+TAC group; ##, $P<0.01$ compared to WT+TAC group; ###, $P<0.001$ compared to WT+TAC group.

Table S7. List of PCR primers

Gene	Primer sequence
rno-HIPK1-Forward	CAGCATCAGCCAATCATC
rno-HIPK1-Reverse	ATTAGACCTCGCCTTCAG
rno-cTnI-Forward	ATGGCGGATGAGAGCAG
rno-cTnI -Reverse	GTTTTCTGGAGGCGGAGA
rno-cTnT-Forward	GCCATCGACCACCTGAATGA
rno-cTnT-Reverse	TTTGGCCTTCCCACGAGTTT
rno-ANP-Forward	GAGCAAATCCCGTATACAGTGC
rno-ANP-Reverse	ATCTTCTACCGGCATCTTCTCC
rno-BNP-Forward	CTGCTTGCGGAGGCGAGAC
rno-BNP-Reverse	TGTTCTGGAGACTGGCTAGGACTTC
rno-β-MHC-Forward	TCCAGCTGAAAGCAGAAAGAG
rno-β-MHC-Reverse	CCTGGGAGATGAACGCATAAT
rno-Col1a1-Forward	GAGCGGAGAGTACTGGATCGA
rno-Col1a1-Reverse	TGCCATTGCTGGAGTTGGA
rno-Col3a1-Forward	TGCCATTGCTGGAGTTGGA
rno-Col3a1-Reverse	GAAGACATGATCTCCTCAGTGTTGA
rno-C/EBPβ-Forward	CAAGCTGAGCGACGAGTACAA
rno-C/EBPβ-Reverse	ACAGCTGCTCCACCTTCTTCT
rno-CREB-Forward	TTCCACTTCTGCCCTCAC
rno-CREB-Reverse	GGTCTCCTCATGGTTCCTG
rno-skeletal α-actinin-Forward	GGTTATGCCCTGCCACA
rno-skeletal α-actinin-Reverse	GCGCACAATCTCACGTTC
rno-cardiac α-actinin-Forward	CCAACCGTGAGAAGATGAC
rno-cardiac α-actinin-Reverse	GTCGCCAGAATCCAGAAC
rno-α-MHC-Forward	CAGCTCAGTCAGGCCAATAGAA
rno-α-MHC-Reverse	CGTTCCTACACTCCTGCACC
rno-SERCA2a-Forward	TGGCAGCAGACTTAGGAAA
rno-SERCA2a-Reverse	AGGGAGTGGCAAACCAA
mmu-HIPK1-Forward	AATCTTCCTGCTTACGGAC
mmu-HIPK1-Reverse	CTCACTCTTTCTCTTCAATCC
mmu-ANP-Forward	AGCCGTTCGAGAACTTGTCTT
mmu-ANP-Reverse	CAGGTTATTGCCACTTAGGTTCA
mmu-BNP-Forward	GAGTCCTTCGGTCTCAAGGC
mmu-BNP-Reverse	TACAGCCCAAACGACTGACG
mmu-β-MHC-Forward	CTTCAACCACCACATGTTCG
mmu-β-MHC-Reverse	TCTCGATGAGGTCAATGCAG
mmu-Col1a1-Forward	CAGAGGCGAAGGCAACA
mmu-Col1a1-Reverse	GTCCAAGGGAGCCACATC
mmu-Col3a1-Forward	AGAACCTGGCCGAGATG
mmu-Col3a1-Reverse	TGGACTTCCGGGCATAC
mmu-CTGF-Forward	TAAGACCTGTGGGATGGG
mmu-CTGF-Reverse	GCAGCCAGAAAGCTCAA
mmu-C/EBPβ-Forward	GCCAACTTCTACTACGAGCCC

mmu-C/EBPβ-Reverse	ACTCGTCGCTCAGCTTGTC
mmu-CREB-Forward	AAAACTCCTAAACCCCAAGC
mmu-CREB-Reverse	GCCTACCATCAAAACACATCT
mmu-skeletal α-actinin-Forward	AAGACGAGACCACCGCTCT
mmu-skeletal α-actinin-Reverse	ACACCGTCCCCAGAATCCAAC
mmu-cardiac α-actinin-Forward	ATGTGTGACGACGAGGAGAC
mmu-cardiac α-actinin-Reverse	GCCATGTTCAATGGGGTACT
mmu-α-MHC-Forward	CAGAGAACGGGGAGTTGG
mmu-α-MHC-Reverse	CTTGGCCTTGCCTTCCT
mmu-SERCA2a-Forward	GGCAGGATGAGGATGTGA
mmu-SERCA2a-Reverse	CTGGGCTGAAGGGCTTA
rno/mmu-18s-Forward	TCAAGAACGAAAGTCGGAGG
rno/mmu-18s-Reverse	GGACATCTAAGGGCATCAC
